# A histone deacetylase 3 and mitochondrial complex I axis regulates toxic formaldehyde production

**DOI:** 10.1126/sciadv.adg2235

**Published:** 2023-05-17

**Authors:** Niek Wit, Ewa Gogola, James A. West, Tristan Vornbäumen, Rachel V. Seear, Peter S. J. Bailey, Guillermo Burgos-Barragan, Meng Wang, Patrycja Krawczyk, Daphne H. E. W. Huberts, Fanni Gergely, Nicholas J. Matheson, Arthur Kaser, James A. Nathan, Ketan J. Patel

**Affiliations:** ^1^ Cambridge Institute of Therapeutic Immunology & Infectious Disease (CITIID), Jeffrey Cheah Biomedical Centre, Cambridge Biomedical Campus, Department of Medicine, University of Cambridge, Cambridge CB2 0AW, UK.; ^2^ MRC Laboratory of Molecular Biology, Francis Crick Avenue, Cambridge CB2 0QH, UK.; ^3^ MRC Weatherall Institute of Molecular Medicine, University of Oxford, John Radcliffe Hospital, Oxford OX3 9DS, UK.; ^4^ Department of Medicine, Weill Cornell Medicine, New York, NY, USA.; ^5^ Department of Haematology, University of Cambridge, Cambridge, UK.; ^6^ Wellcome-MRC Cambridge Stem Cell Institute, Jeffrey Cheah Biomedical Centre, University of Cambridge, Cambridge, UK.; ^7^ Cancer Research UK Cambridge Institute, Li Ka Shing Centre, University of Cambridge, Cambridge, UK.; ^8^ Department of Biochemistry, University of Oxford, Oxford, UK.; ^9^ NHS Blood and Transplant, Cambridge, UK.; ^10^ Division of Gastroenterology and Hepatology, Department of Medicine, University of Cambridge, Cambridge, UK.

## Abstract

Cells produce considerable genotoxic formaldehyde from an unknown source. We carry out a genome-wide CRISPR-Cas9 genetic screen in metabolically engineered HAP1 cells that are auxotrophic for formaldehyde to find this cellular source. We identify histone deacetylase 3 (HDAC3) as a regulator of cellular formaldehyde production. HDAC3 regulation requires deacetylase activity, and a secondary genetic screen identifies several components of mitochondrial complex I as mediators of this regulation. Metabolic profiling indicates that this unexpected mitochondrial requirement for formaldehyde detoxification is separate from energy generation. HDAC3 and complex I therefore control the abundance of a ubiquitous genotoxic metabolite.

## Teaser

Genome-wide screens uncover the regulation of endogenous toxic formaldehyde production in human cells.

## INTRODUCTION

Formaldehyde, the simplest reactive aldehyde, is an established common industrial pollutant and carcinogen (*1*). However, recent discoveries indicate that we produce a large amount of this profoundly toxic molecule within our bodies, with levels as high as 40 μM detected in murine blood (*2*). Protection against endogenous formaldehyde is essential because of its ability to cause irreversible cellular damage. This is provided by a two-tiered protection system that consists of the enzymes alcohol dehydrogenase 5 (ADH5) and aldehyde dehydrogenase 2 (ALDH2) that detoxify this aldehyde (Tier-1) (*2*, *3*) and two DNA repair pathways: Fanconi anemia and transcriptional coupled nucleotide excision repair (Tier-2) that repair DNA damage caused by formaldehyde (*4*, *5*). Genetic loss of either Tier-1 or Tier-2 protection results in profound human phenotypes causing developmental defects, cachexia, premature aging, loss of blood production, and cancer predisposition (*4*, *5*).

While we understand how we are protected against formaldehyde, a fundamental question is to determine its cellular sources and regulators. A small amount of formaldehyde originates from the oxidative decomposition of folic acid derivatives, but the concentration of this vitamin is several magnitudes lower than that of blood formaldehyde (*6*). Enzymatic oxidative demethylation from a class of histone and nucleic acid demethylases releases formaldehyde (*7*), but the extent to which this source contributes to the total formaldehyde burden is not known. Formaldehyde is also hardwired into cellular metabolism, as its detoxification by ADH5 and ALDH2 creates formate, which is a key substrate for one-carbon (1C) metabolism (*6*). This supply of 1C units is independent from the dominant serine source (*8*) and, in one instance, can produce enough formate to sustain cellular 1C requirements (*6*). Here, we exploit this property of cellular formaldehyde metabolism to perform genetic screens to identify regulators and sites of formaldehyde production. We discover that the histone deacetylase 3 (HDAC3) regulates formaldehyde production and that its detoxification requires a noncanonical function of mitochondrial complex I.

## RESULTS

### A CRISPR-Cas9 mutagenesis screen identifies HDAC3 as a strong suppressor of endogenous formaldehyde

**Fig. 1.**
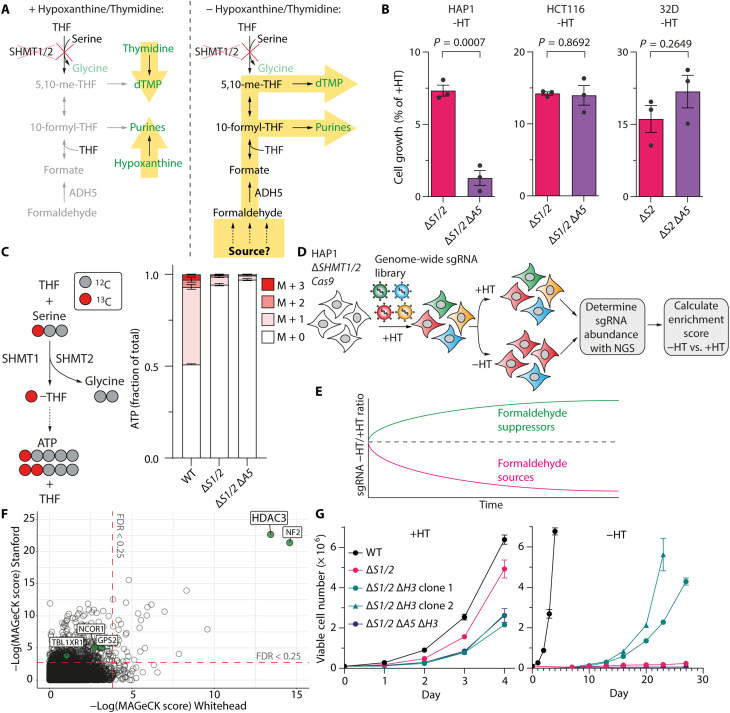
CRISPR-Cas9 forward genetic screening identifies histone deacetylase 3 (HDAC3) as a formaldehyde suppressor. (**A**) A schematic depicting one-carbon (1C) metabolism and the contribution of endogenous formaldehyde to 1C metabolism in serine hydroxymethyltransferase (SHMT)–deficient cells. (**B**) Growth of HAP1, HCT116, and 32D strains ±HT (means ± SEM, *n* = 3; 3 days −HT). (**C**) Metabolic tracing strategy to determine serine cleavage activity by SHMT enzymes using [3-^13^C]serine (left). Fractional isotopic labeling of adenosine triphosphate (ATP) in HAP1 cells fed [3-^13^C]serine (means ± SEM, *n* = 3; 24 hours −HT) (right). (**D**) A schematic of CRISPR-Cas9 screen to identify formaldehyde suppressors using Δ*SHMT1/2* (Δ*S1/2*) *Cas9* cells. sgRNA, single guide RNA. (**E**) Predicted outcomes of formaldehyde regulator screens. (**F**) Results of genome-wide formaldehyde suppressor screens in HAP1 ∆*S1/2 Cas9* cells. (**G**) Growth curves of HAP1 strains ±HT (means ± SEM, *n* = 3). *P* values were determined by two-tailed *t* tests.

We first established a model cell system that is reliant on endogenous formaldehyde as a 1C donor for growth based on our previous work (*6*). Deletion of the serine hydroxymethyltransferase enzymes (*SHMT1 *and *SHMT2*), which cleave serine to glycine (*8*), shuts off the predominant source of formate in cells. This genetic situation creates a dependence on formate derived from formaldehyde for 1C units and nucleotide synthesis, which we could now exploit to find genetic regulators for this dependency (Fig. 1A). We then identified cell lines where this SHMT-independent reliance on formate for growth could be blocked by deletion of *ADH5*, the main enzyme that converts formaldehyde to formate. Such predicted dependency should only be restored by exogenous nucleotide supplementation with hypoxanthine and thymidine (HT). Of several cell lines tested, only HAP1 cells displayed further loss of viability when 1C metabolism genes *SHMT1* and *SHMT2* (Δ*S1/2*) and *ADH5* (Δ*A5*) were simultaneously deleted (Fig. 1B and fig. S1, A to D). Metabolic characterization of this HAP1 Δ*S1/2* strain and other strains confirmed its lack of canonical SHMT activity using [3-^13^C]serine (Fig. 1C and fig. S1F), despite normal serine uptake (fig. S1E), and its ability to use formaldehyde as a 1C donor in an ADH5-dependent manner (fig. S1G). HAP1 Δ*S1/2* cells also showed a defect in cell cycle progression when nucleotide synthesis is impaired, accumulating in G_1_ phase after the withdrawal of HT (fig. S1, H to J).

Having established HAP1 Δ*S1/2* cells as a suitable model to screen for sources or regulators of endogenous formaldehyde, we performed parallel genome-wide CRISPR-Cas9 knockout screens using two independent single guide RNA (sgRNA) libraries (Fig. 1, D and E) (*9*, *10*). Screens were undertaken with and without HT supplementation to control for conditions where the Δ*S1/2* 1C metabolism deficiency is bypassed by exogenous nucleotide precursors (Fig. 1, A and E). We focused on genes that might act to suppress formaldehyde production because loss of these genes in Δ*S1/2* cells would exhibit faster growth in HT-deficient but not HT-proficient conditions (Fig. 1E and fig. S2A). Reassuringly, the strength of this genetic selection drives mutations, which result in the reactivation of SHMT2 at later time points (fig. S2, B and C). We therefore focused on early restoration of cell growth and identified two genes that were highly enriched for sgRNAs in growth conditions without but not with HT: *HDAC3* and neurofibromin 2 (*NF2*; also known as Merlin) (Fig. 1F). Key components of the HDAC3 histone deacetylation complex [transducin beta-like 1X-related protein 1 (*TBL1XR1*), G protein pathway suppressor 2 (*GPS2*), and nuclear receptor corepressor 1 (*NCOR1*)] (*11*) were also enriched in the screens (Fig. 1F and fig. S2, D and E), but not other members of the wider HDAC family (fig. S2F). This is consistent with the possibility that the HDAC3 complex functions to suppress formaldehyde production. We validated the screen by demonstrating a notable increase in cell growth in Δ*S1/2* cells deficient in HDAC3 (Δ*S1/2* Δ*H3*) when exogenous nucleotides were withdrawn (Fig. 1G and fig. S2G), with an expected increase in the proportion of cells cycling in S-G_2_-M phases (fig. S2H). Growth suppression was restored by complementation of FLAG-HDAC3 in Δ*S1/2* Δ*H3* cells (fig. S2, I and J). The HDAC3 loss–dependent growth enhancement was not observed in 1C-proficient cells and was not due to the restoration of SHMT1/SHMT2 expression (fig. S2, K to M). HDAC3 loss only restored growth in an ADH5-proficient manner in the absence of nucleotides (Fig. 1G). Thus, HDAC3 is a potent suppressor of growth in cells dependent on ADH5-derived 1C units (Fig. 1A).

### Loss of HDAC3 function increases de novo purine synthesis activity and kills BRCA-deficient tumor cells

**Fig. 2.**
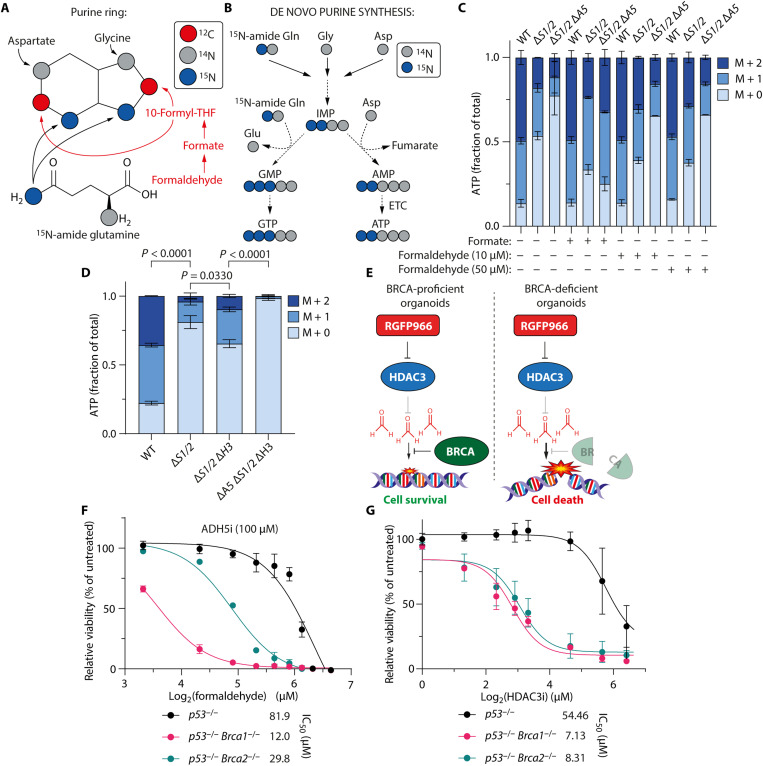
Loss of histone deacetylase 3 (HDAC3) function increases de novo purine synthesis activity and kills BRCA-deficient tumor cells. (**A**) Metabolic origins of purine ring atoms. (**B**) Metabolic tracing strategy to determine de novo purine synthesis activity using [^15^N-amide]glutamine. (**C**) Fractional isotopic labeling of adenosine triphosphate (ATP) in HAP1 strains fed [^15^N-amide]glutamine (means ± SEM, *n* = 3) ±formate (400 μM) or formaldehyde (7 days −HT). WT, wild-type. (**D**) Fractional isotopic labeling of ATP in HAP1 strains fed [^15^N-amide]glutamine (means ± SEM, *n* = 3; 7 days −HT). (**E**) Synthetic lethal interaction between BRCA deficiency and HDAC3 inhibition. (**F**) In vitro responses of BRCA-proficient (*p53*^−/−^) and BRCA-deficient (*p53*^−/−^ *Brca1*^−/−^ and *p53*^−/−^ *Brca2*^−/−^) organoids to formaldehyde and alcohol dehydrogenase 5 (ADH5) inhibition (N6022). Representative of three independent repeats (means ± SEM, *n* = 3). (**G**) In vitro responses of BRCA-proficient and BRCA-deficient organoids to RGFP966 (HDAC3 inhibitor). Representative of three independent repeats (means ± SEM, *n* = 3). *P* values were determined by pairwise chi-square tests of average distributions. IC_50_, median inhibitory concentration.

While current means lack sensitivity to detect endogenous formaldehyde directly, it is possible to use metabolic tracing to determine whether HDAC3 suppresses endogenous formaldehyde. To achieve this, we measured the incorporation of [^15^N-amide]glutamine into de novo purine synthesis (Fig. 2, A and B). SHMT deficiency constrains the 1C cycle to rely on formaldehyde and supply two carbon atoms to each purine ring via 10-formyltetrahydrofolate (10-formyl-THF; Figs. 1A and 2A) (*6*). The incorporation of glutamine-derived ^15^N into a purine ring is therefore directly proportional to the availability of 10-formyl-THF.

We validated this approach in HAP1 wild-type (WT), Δ*S1/2*, and Δ*S1/2* Δ*A5* cells with or without formate or formaldehyde supplementation. As expected, Δ*S1/2* cells showed decreased ^15^N incorporation into adenosine triphosphate (ATP) compared to the WT, which was further reduced in Δ*S1/2* Δ*A5* cells (Fig. 2C), consistent with endogenous formaldehyde detoxification driving purine synthesis. Supplying 1C units by formate treatment restored ^15^N incorporation into ATP in both HAP1 Δ*S1/2* and Δ*S1/2* Δ*A5* cells, similarly to WT levels. Formaldehyde treatment only restored de novo purine synthesis in the ADH5-proficient HAP1 Δ*S1/2* cells (Fig. 2C), confirming that 1C units are supplied in an ADH5-dependent manner.

Having established that ^15^N incorporation into ATP directly relates to formaldehyde detoxification in HAP1 Δ*S1/2* cells, we tested whether HDAC3 loss increased de novo purine synthesis and whether this was dependent on ADH5-derived 10-formyl-THF (Fig. 2, A to D). HAP1 Δ*S1/2* cells showed a threefold reduction compared to WT cells in purine synthesis, measured by ^15^N incorporation into ATP (Fig. 2D), consistent with the growth defect observed when no nucleotides are supplemented (Fig. 1B). *HDAC3* deletion in Δ*S1/2* cells doubled purine synthesis rates, and this was entirely dependent on ADH5 (Fig. 2D). The effect of *HDAC3* loss was not due to differences in apoptosis, mitochondrial content, or nucleotide salvage pathway activity (fig. S3, A to N), indicating that HDAC3 controls the supply of formaldehyde for 1C units in an ADH5-dependent manner (Fig. 2D).

If HDAC3 regulates the use of endogenous formaldehyde for 1C metabolism, then cells that are sensitive to formaldehyde-induced DNA damage should be more susceptible to HDAC3 inhibition. Therefore, we made use of breast cancer 1/2 (BRCA1/2) and p53-deficient mouse mammary tumor organoids as a formaldehyde-sensitive and clinically relevant model (Fig. 2E) (*12*). Formaldehyde sensitivity was demonstrated by inhibiting ADH5 to prevent Tier-1 detoxification and comparing cell death in *p53^−/−^* tumor organoids to those with combined p53 and BRCA deficiencies (*Brca1^−/−^* or *Brca2^−/−^*; Fig. 2F). BRCA loss, particularly *Brca1^−/−^*, resulted in increased tumor death following treatment with exogenous formaldehyde (Fig. 2F). We next treated *p53^−/−^* BRCA1/2-proficient or BRCA1/2-deficient tumor organoids with the highly specific HDAC3 inhibitor (RGFP966) (*13*). HDAC3 inhibition markedly increased cell death in BRCA1/2-deficient tumors compared to the *p53^−/−^* control (Fig. 2G). This was not due to altered levels of HDAC3 or ADH5 (fig. S3O). We also verified that HDAC3 inhibition enhanced growth in HAP1 Δ*S1/2* cells, in an ADH5-dependent manner, similarly to HDAC3 loss (fig. S3P), providing further evidence for the enzymatic activity of HDAC3 suppressing the formation of endogenous formaldehyde.

### Mitochondrial complex I is coessential with HDAC3 loss in formaldehyde auxotrophy

**Fig. 3.**
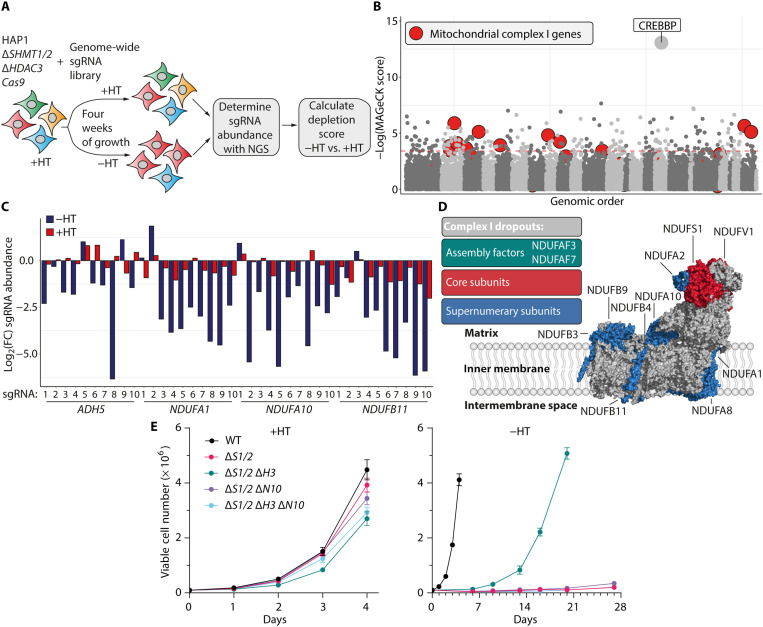
CRISPR-Cas9 screening reveals components of mitochondrial complex I as coessential with histone deacetylase 3 (HDAC3) loss. (**A**) CRISPR-Cas9 screen to identify formaldehyde sources or regulators using Δ*SHMT1/2* Δ*HDAC3* (Δ*S1/2* Δ*H3*) cells. (**B**) Results of formaldehyde source CRISPR-Cas9 screen in HAP1 ∆*S1/2* Δ*H3 Cas9* cells using the Stanford whole-genome single guide RNA (sgRNA) library (*9*). (**C**) Depletion of alcohol dehydrogenase 5 (ADH5) and complex I sgRNAs without hypoxanthine and thymidine (HT) . Values indicate log_2_-transformed fold change in sgRNA abundance compared to plasmid. (**D**) Classification of complex I dropouts and their location in the complex (*18*). (**E**) Growth curves of HAP1 strains with ±HT (means ± SEM, *n* = 3).

The identification of HDAC3 as a suppressor of endogenous formaldehyde raised questions as to the source or regulation of the 1C units that supported growth in the Δ*S1/2* cells. To address this, we performed a genome-wide CRISPR-Cas9 drop-out screen in Δ*S1/2* Δ*H3* cells (Fig. 3, A to C, and fig. S4A) to functionally identify genes that regulate the formaldehyde supply of 1C units downstream of HDAC3. We predicted that sgRNA targeting genes involved in formaldehyde production would be lost over time in growth conditions without HT but not with HT (Figs. 1E and 3A). Cyclic adenosine monophosphate (cAMP) response element–binding protein (CREB) binding protein (*CREBBP*), a transcriptional activator with lysine acetyltransferase activity (*14*), was the strongest hit by several orders of magnitude (Fig. 3B). This provided further validation of a functional role for HDAC3 in formaldehyde regulation, as CREBBP-mediated acetylation would antagonize deacetylation by HDAC3 (*15*, *16*). Expectedly, we also observed depletion of sgRNAs targeting ADH5 (Fig. 3C), confirming the dependency on 1C units derived from formaldehyde.

We next undertook a bioinformatic pathway analysis to find the most enriched cellular processes associated with sgRNA loss of representation (fig. S4B). This analysis yielded processes involving mitochondrial complex I within the mitochondrial respiratory chain as top hits, with 12 complex I genes dropping out in the screen without HT supplementation (Fig. 3D). Complex I consists of over 40 subunits, of which 14 are core subunits (*17*). The complex I genes that dropped out in the screen did not cluster to a particular location within the membrane-embedded complex (Fig. 3D) (*18*), suggesting that the entire complex is required to maintain the growth of HAP1 Δ*S1/2* Δ*H3 Cas9* cells.

We validated the screen results by deleting the top-ranked complex I gene, reduced form of nicotinamide adenine dinucleotide (NADH):ubiquinone oxidoreductase subunit A10 (*NDUFA10*; further abbreviated as *N10*), in HAP1 Δ*S1/2* and Δ*S1/2* Δ*H3* mutants (fig. S4C). In the presence of HT, *NDUFA10* deletion caused a mild growth defect, irrespective of the genotype (Fig. 3E and fig. S2, K and L). Upon withdrawal of HT, Δ*S1/2* Δ*H3* Δ*N10* mutants failed to grow (Fig. 3E). The growth of the Δ*S1/2* mutants without HT supplementation was not altered by *NDUFA10* deletion (Fig. 3E).

This requirement for complex I was likely independent of its canonical activity, as treatment with metformin, a complex I inhibitor (*19*), did not affect the growth of HAP1 Δ*S1/2* Δ*H3* mutants (fig. S4D). Metformin reduced the respiratory capacity in HAP1 cells (fig. S4, E and F), and HAP1 cells should have normal metformin uptake (fig. S4G) (*20*). Therefore, complex I is essential for the growth of the combined *SHMT*/*HDAC3* mutants, but this is unlikely to be directly related to its canonical enzymatic activity.

### Mitochondrial complex I supports formaldehyde detoxification

**Fig. 4.**
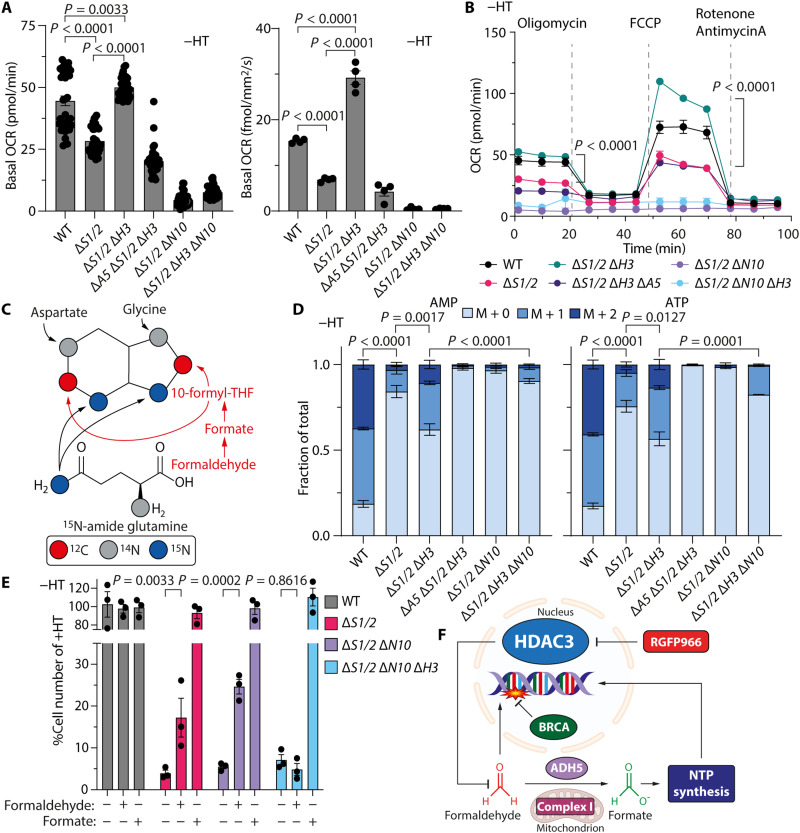
Mitochondrial complex I supports formaldehyde detoxification. (**A**) Basal oxygen consumption rate (OCR) without hypoxanthine and thymidine (HT) (7 days) of various HAP1 genotypes obtained with a Seahorse XFe96 Analyzer (means ± SEM, *n* ≥ 12, technical replicates, representative experiment shown) (left). Basal OCR of HAP1 strains obtained with a Resipher OCR analyzer. Representative experiment shown (means ± SEM, *n* = 4, technical replicates; 8 hours −HT) (right). (**B**) Bioenergetic assays of OCR in HAP1 strains in growth medium without HT (7 days; means ± SEM, *n* = > 12, technical replicates, representative experiment shown). (**C**) Metabolic origins of purine ring atoms. (**D**) Fractional isotopic labeling of adenosine monophosphate (AMP) (left) and adenosine triphosphate (ATP) (right) in HAP1 strains fed [^15^N-amide]glutamine (means ± SEM, *n* = 4; 7 days −HT). (**E**) Cell growth of HAP1 strains in media ± sodium formate (400 μM) or formaldehyde (15 μM; means ± SEM, *n* = 3; 3 days −HT). (**F**) Model for formaldehyde generation by mitochondrial complex I and its suppression by histone deacetylase 3 (HDAC3). *P* values were determined by one-way analysis of variance (ANOVA) (A and B), two-way ANOVA (E), or pairwise chi-square tests of average distributions (D).

To further understand how complex I was required for the HDAC3 loss–mediated regulation of growth, we performed a systematic evaluation of metabolic adaption to complex I loss in the Δ*S1/2* Δ*H3* mutants. No change in complex I transcript levels were observed following *HDAC3* loss (fig. S4H) (*21*). However, bioenergetic analyses, using both Seahorse flux analysis and a Resipher, demonstrated a marked increase in respiratory capacity following *HDAC3* loss, which was completely ablated by *NDUFA10* or *ADH5* deletion (Fig. 4, A and B, and fig. S4, I to K). *HDAC3* deletion in SHMT-proficient cells did not enhance the maximal respiratory capacity (fig. S4, L and M), and as expected, deletion of *NDUFA10* shut down oxygen consumption (Fig. 4, A and B, and fig. S4, I to K).

Growth in medium containing HT or formate restored the reduced respiratory capacity of Δ*S1/2* and Δ*S1/2* Δ*H3* Δ*A5* mutant cells (fig. S5, A to D), as did formaldehyde treatment with the Δ*S1/2* strain (fig. S5, E to G). Therefore, while the enhanced respiratory capacity in Δ*S1/2* Δ*H3* mutants is likely related to de novo nucleotide synthesis, it is also possible that the changes in oxygen consumption simply correlate with the restoration of cell growth.

To further dissect the contribution of complex I to the supply of 1C units following HDAC3 loss, we examined the metabolic rewiring following complex I deficiency in the SHMT-deficient background using gas chromatography–mass spectrometry (GC-MS) analysis and liquid chromatography–mass spectrometry (LC-MS). We first measured whether HDAC3 loss altered tricarboxylic acid (TCA) cycle function, as complex I regenerates the nicotinamide adenine dinucleotide pool from NADH produced by the cycle (fig. S6A) (*22*). HAP1 Δ*S1/2* mutants displayed alterations in TCA cycle metabolites and related amino acids that were consistent with reduced succinate dehydrogenase activity (fig. S6B) (*23*), and similar patterns were previously observed in SHMT2 or MTHFD2 mutant cells (fig. S6C) (*24*, *25*). However, HAP1 Δ*S1/2* Δ*H3* mutants showed similar changes in relative metabolite abundance to Δ*S1/2* cells alone (fig. S6B), indicating that TCA metabolite levels could not explain the complex I phenotype in the Δ*S1/2* Δ*H3* cells. Similarly, TCA metabolic flux analysis using [^15^N_2_^13^C_5_]glutamine labeling (fig. S6A) (*26*), showed a reduction in both oxidative metabolism and reductive carboxylation in the Δ*S1/2* cells, but a further deletion of *HDAC3* did not influence this (fig. S6, D to K).

Having excluded a change in TCA cycle flux responsible for the formaldehyde-dependent growth, we determined whether complex I was required for de novo nucleotide synthesis under conditions of *HDAC3* loss by labeling newly formed adenosine monophosphate (AMP) and ATP with [^15^N-amide]glutamine. HAP1 Δ*S1/2* Δ*H3* mutants showed an increase in the fraction of newly synthesized AMP and ATP (Fig. 4, C and D) as previously observed (Fig. 2D). Notably, genetic ablation of *NDUFA10* shut down nucleotide synthesis and markedly reduced the fractional labeling of both AMP and ATP in the Δ*S1/2* Δ*H3* (Fig. 4D). Therefore, complex I was required to supply 1C units for purine synthesis in Δ*S1/2* Δ*H3* cells.

Last, by supplementing Δ*S1/2* Δ*H3* Δ*N10* cells with formate or formaldehyde (Fig. 4E), we determined whether complex I was either the source of formaldehyde or is required for its detoxification. Formate completely restored growth in the Δ*S1/2* Δ*H3* Δ*N10* mutant to a comparative level in WT HAP1 cells, indicating that 1C metabolism was intact and sufficient to maintain normal growth in complex I deficiency (Fig. 4E). As expected, exogenous formaldehyde also restored growth of Δ*S1/2* and Δ*S1/2* Δ*N10* cells (Fig 4E). However, growth of Δ*S1/2* Δ*H3* Δ*N10* cells could not be enhanced by formaldehyde. Together, these findings confirm that complex I is required to detoxify formaldehyde, which can be used to sustain nucleotide synthesis under conditions of SHMT deficiency (Fig. 4F).

## DISCUSSION

Formaldehyde is produced at substantial levels in cells, but despite speculation, it was not known where much of it is generated. This question has been difficult to address because of the limitations in detecting endogenous formaldehyde. Our creation of a cell line that is auxotrophic for endogenous formaldehyde opens the way for unbiased genetic approaches to address this and reveals that HDAC3 suppresses the production of cellular formaldehyde. How precisely HDAC3 acts to suppress formaldehyde and the biochemical basis by which altered complex I activity controls this is not clear. Our finding that formaldehyde detoxification by complex I is likely distinct from its energy-producing activity may define an unknown activity for this important conserved protein complex in biology. This is supported by the observation that metformin treatment improves blood production and reduces tumor susceptibility in a murine model of Fanconi anemia likely through scavenging aldehydes rather than its direct action on the electron transport chain (*27*).

Our findings indicate that HDAC3 drastically suppresses the levels of formaldehyde exposure. This reveals that in addition to the two-tier protection mechanism against this aldehyde, HDAC3 suppression provides yet another additional layer of protection. A potential benefit of removing this protection is in BRCA-deficient cancers that are unable to repair such damage because they lack Tier-2 protection. Pharmacological inhibition of HDAC3 is cytotoxic to BRCA-deficient murine cancer organoids, an established model system for analogous human cancers (*12*). It will be important to establish whether this has therapeutic potential.

Formaldehyde genotoxicity is an important driver for the phenotypes of two rare diseases: Fanconi anemia and aldehyde degradation deficiency syndrome (*2*, *4*, *28*). Stimulating HDAC3 activity might provide a means to suppress cellular formaldehyde production and thereby limit the DNA damage driving these illnesses. The involvement of complex I may also reduce genotoxicity. In this regard, it is provocative to note that down-regulation of complex I in mouse oocytes helps preserve them by limiting the production of reactive oxygen species (*29*), but in the context of Fanconi anemia, where complex I defects have been reported, this may contribute to genotoxicity and explain the infertility seen in the disease (*30*).

## MATERIALS AND METHODS

### Constructs

To overexpress 3xFLAG-HDAC3, the human cDNA sequence of HDAC3 (NCBI CCDS4264.1) was synthesized (GeneArt Gene Synthesis) with an N-terminal 3xFLAG sequence. This sequence was flanked by an Nhe I restriction site and Kozak sequence at the 5′ end and by an Eco RV restriction site at the 3′ end. Next, this was cloned (Nhe I/Eco RV digested) into pExpress (Nhe I/Eco RV digested) (*31*). Last, the resulting vector was then digested with Spe I and the fragment containing the β-actin promoter, 3xFLAG-HDAC3, and SV40 transcription termination site was subsequently cloned into plox-BSR (Spe I digested) (*31*) and sequence-verified.

The cell cycle reporter, henceforth F3C, was cloned as follows: First, an oligo (Oligo-pAAVS1-Nst-MCS_polyA) containing the β-globin polyadenylate [poly(A)] signal flanked by 5′ Spe I/3′ Sal I sites was cloned into the Spe I/Sal I–digested adeno-associated virus integration site 1 (AAVS1) targeting vector pAAVS1-Nst-MCS (gift from K. Woltjen; Addgene, #80487). This oligo also contained upstream of the poly(A) signal, Asc I and Not I sites. The resulting vector is henceforth the host vector.

Next, a separate donor vector (pMA-pCAG-delSalI-kozak-F3v3-Cdt1) was cloned that contains the cell cycle reporter sequence. This reporter contains a synthetic promoter [cytomegalovirus (CMV) enhancer, followed by the chicken β-actin promoter and a chimeric sequence of chicken β-actin and rabbit β-globin introns]. This promoter drives the expression of a polycistronic transcript containing coding sequences for histone H2B-miRFP670, P2A, mCherry-hCdt1(1/100)Cy(−), T2A, and EGFP-hGEM(1-110). Upstream of the synthetic promoter is an Asc I site, and downstream of the polycistronic transcript is a Not I site, which are used for cloning the reporter sequence of the final F3C into the host vector. Each of the elements described above was gene-synthesized separately (GeneArt Gene Synthesis) and assembled using restriction and Gibson cloning to form the complete donor vector.

### Cell lines and reagents

Human embryonic kidney (HEK) 293T cells (a gift from the Larrieu laboratory, Cambridge Institute for Medical Research) used for lentivirus production were cultured in Dulbecco’s modified Eagle medium (DMEM; Gibco) containing 10% fetal bovine serum (FBS; Sigma-Aldrich) and penicillin-streptomycin (Gibco).

WT HAP1 cells (near-haploid human cell line derived from the KBM-7 chronic myelogenous leukemia) were purchased from Horizon and cultured in Iscove’s modified Dulbecco’s medium (Gibco) supplemented with 10% dialyzed serum (Sigma-Aldrich) and penicillin-streptomycin (Gibco) with or without hypoxanthine (100 μM) and thymidine (16 μM) supplement (Gibco), as indicated.

HAP1 ∆*S1/2* and ∆*S1/2* ∆*A5* cells were generated by CRISPR-Cas9–mediated knockout of SHMT2 in SHMT1-deficient cells or SHMT1/ADH5 double-deficient cells, respectively, as described in (*6*). Briefly, cells were transfected (according to standard procedures; TurboFectin 8.0, OriGene) with a plasmid expressing the relevant sgRNA and Cas9 (pX458, gift from F. Zhang), and 2 days after transfection, green fluorescent protein–positive cells were single cell–sorted into 96-well plates in medium with HT and 20% FBS using an FACSAria III cell sorter (BD Biosciences). After 2 weeks, clones were selected and analyzed by immunoblotting for depletion of the target protein (described below). Deletion of HDAC3 and NDUFA10 was achieved in a similar fashion in existing HAP1 genotypes.

Murine myeloid 32D cells were a gift from A. Green (Department of Haematology, Cambridge) and maintained in RPMI 1640 containing 10% dialyzed serum, penicillin-streptomycin, and interleukin-3 (5 ng/ml; PeproTech). Knockout strains were generated using CRISPR-Cas9: Cells were transfected with pX458 containing an sgRNA targeting *Shmt2* or an sgRNA pair targeting *Adh5* in pX461 (gift from F. Zhang) using the Amaxa Nucleofector reagent according to standard procedures. Knockout clones were obtained as described above.

HCT116 strains (human colon cancer cell line) were a gift from J. D. Rabinowitz (*24*, *32*). *ADH5* was deleted in *SHMT1/2* double knockouts using CRISPR-Cas9: Cells were transfected using FuGENE (Promega), according to standard procedures, with a plasmid expressing the *ADH5* sgRNA and Cas9 (pX458, gift from F. Zhang). Knockout clones were obtained as described above.

To complement HDAC3 in HAP1 ∆*S1/2* ∆*H3* cells, cells were seeded in six-well plates with HT supplement. One day after plating, 2 μg of the 3xFLAG-HDAC3 construct was transfected using 6 μl of TurboFectin 8.0, according to standard procedures. Two days after transfection, cells were selected with blasticidin (20 μg/ml; Gibco) for at least 7 days. Clones were obtained as described above.

HAP1 cell cycle reporter (F3C) AAVS1 knock-in strains were generated by cotransfection of the F3C reporter plasmid and the AAVS1 sgRNA and Cas9 expressing plasmid pXAT2 (Addgene, 80494). Transfection was mediated by TurboFectin 8.0 (OriGene), according to standard procedures. Two days after transfection, cells were selected with Geneticin (Formedium Ltd.) at 4 mg/ml for at least 7 days. To obtain clones, cells were single cell–sorted on a FACSAria III cell sorter (BD Biosciences) into 96-well plates in medium with HT and 20% FBS. Clones were obtained as described above.

Established mouse mammary tumor organoid lines were a gift from J. Jonkers, Netherlands Cancer Institute, Amsterdam, Netherlands. Organoids were grown as described previously (*12*). Briefly, organoid cultures were grown embedded in 40-μl droplets of Cultrex Reduced Growth Factor Basement Membrane Extract Type 2 (BME, Trevigen), in a 24-well suspension plates and in Advanced DMEM/F12 media (Gibco) supplemented with 1 M Hepes (Sigma-Aldrich), GlutaMAX (Invitrogen), penicillin-streptomycin (Gibco), B27 (Gibco), 125 μM *N*-acetyl-l-cysteine (Sigma-Aldrich), and murine epidermal growth factor (50 ng/ml; Invitrogen).

All cells described were grown in a 5% CO_2_ incubator at 37°C. All sgRNA sequences that were used to make knockout strains can be found in table S1.

### Cell growth assays

To determine growth in different growth media, HAP1 cells were seeded in six-well plates (Falcon) at 10^5^ cells per well with or without HT, 400 μM sodium formate (Sigma-Aldrich), or 50 μM formaldehyde (Thermo Fisher Scientific). After 72 hours, trypan blue (Sigma-Aldrich)–negative cells were counted using a hemocytometer. For each condition, the number of trypan blue–negative cells were divided by the number of trypan blue–negative cells of the condition with HT supplement and converted to a percentage.

For growth curves, HAP1 cells were seeded in six-well plates at 10^5^ cells per well with or without HT. At indicated days, trypan blue–negative cells were counted using a hemocytometer. The absolute number of trypan blue–negative (viable) cells was plotted for these time points.

To determine the effect of metformin on cell growth on HAP1 WT and ∆*S1/2* ∆*H3* cells, 10^5^ cells were plated ±HT and ±metformin (Merck). HAP1 WT (±HT) and ∆*S1/2* ∆*H3* +HT were counted after 3 days, whereas ∆*S1/2* ∆*H3* −HT was counted after 14 days.

To test the effect on growth of RGFP966 on HAP1 Δ*S1/2* and Δ*S1/2* Δ*A5* cells, 6 × 10^5^ cells were seeded −HT. One day later, RGFP966 was added, after which the cells were allowed to grow for another 10 days (the medium was replaced every 3 days). After 10 days of RGFP966, the cells were counted as described above.

For organoid cytotoxicity assays, organoids were first dissociated into single cells by resuspending the cultures in TrypLE (Gibco) and incubation at 37°C for 10 min. Cell suspensions were then passed through 40-μm cell strainer to remove cell clamps. A total of 50,000 cells were seeded per well in a 40-μl droplet of BME and cultured for 5 days in drug/inhibitor-containing media. Cell viability was then assessed using resazurin-based CellTiter-Blue assay following the manufacturer’s protocol (Promega). All experiments were performed at least in duplicate and repeated at least three times. The following drugs were used: HDAC3 inhibitor RGFP966 [stock 137.97 mM in dimethyl sulfoxide (DMSO); Stratatech Scientific Ltd.], cisplatin (stock 3.3 mM stock in saline; Cambridge Bioscience Ltd.), olaparib (stock 100 mM in DMSO; Selleckchem), formaldehyde [stock 16% (w/v) methanol-free solution; Thermo Scientific], and ADH5 inhibitor N6022 (stock 50 mM in DMSO; Selleckchem).

### Stable isotope labeling of cells

To determine the capacity of cells to generate 1C units derived from serine, cells were first seeded in six-well plates in medium without HT, after which the cells were labeled with 0.4 mM (HAP1/HCT116) or 0.285 mM (32D) [3-^13^C]serine (Sigma-Aldrich) for 24 hours. To extract metabolites, cells were washed with ice-cold phosphate-buffered saline (PBS), and 200 μl of ice-cold extraction fluid was added [acetonitrile/H_2_OMQ/methanol (ratio, 3:2:5)]. Next, the extract was sonicated in a Bioruptor sonicator (Diagenode) for 10 min (10 cycles of 30 s on and 30 s off) at standard settings. Last, extracts were centrifuged in a chilled tabletop centrifuge at maximum speed for 10 min, and supernatant, without disturbing the pellet, was stored at −80°C.

To determine de novo nucleotide synthesis activity, HAP1 cells were plated at 2 × 10^5^ (WT HAP1), 10^6^ (∆*S1/2* ∆*H3*), or 2 × 10^6^ (all other 1C mutants) cells per well in medium without HT and left to grow for 6 days. At day 6 without HT, medium was aspirated and medium without HT containing 1 mM [^15^N-amide]glutamine (Sigma-Aldrich) was added. After another 24 hours, metabolites were extracted as described above. WT HAP1 cells were split at days 3 and 5.

To determine the effect of formaldehyde or formate on de novo nucleotide synthesis, cells were plated without HT as above and left to grow for 6 days. On day 6, 1 mM [^15^N-amide]glutamine (Cambridge Isotopes Laboratories Inc.), including 400 μM sodium formate (Sigma-Aldrich) or 10 or 50 μM formaldehyde, was added. After another 24 hours, metabolites were extracted as described above. WT HAP1 cells were split at days 3 and 5.

To determine nucleotide salvage activity (*33*), HAP1 cells were plated at 2 × 10^5^ (WT HAP1), 10^6^ (∆*S1/2* ∆*H3*), or 2 × 10^6^ (all other 1C mutants) cells per well in medium without HT and left to grow for 7 days. At day 7 without HT, 3 hours before metabolite extraction, medium was aspirated, and medium without HT containing 1 mM [^15^N_2_,^13^C_5_]glutamine (Sigma-Aldrich) was added. Metabolites were extracted as described above. WT HAP1 cells were split at days 3 and 5.

All solvents used were of LC-MS–grade purity. All stable isotope tracers were added to medium that also contained unlabeled molecules: serine at 0.4 mM and glutamine at 4 mM.

### LC-MS of aqueous metabolites

For MS analysis, a Q Exactive Plus Orbitrap coupled to a Vanquish Horizon ultrahigh-performance LC system was used. Samples were then analyzed using a bridged ethylene hybrid (BEH) amide hydrophilic interaction LC (HILIC) approach for the nucleoside phosphates. For this analysis, the strong mobile phase (A) was aqueous 100 mM ammonium carbonate, and the weak mobile phase was acetonitrile (B) with 1:1 water:acetonitrile being used for the needle wash. The LC column used was the ACQUITY Premier BEH Amide column (150 mm by 2.1 mm, 1.7 μm; Waters). The following linear gradient was used: twenty percent A in acetonitrile for 1.5 min followed by an increase to 60% A over 3.5 min with a further 1 min at 60% A, after which the column was re-equilibrated for 4.0 min. The total run time was 10 min; the flow rate was 0.5 ml/min, and the injection volume was 5 μl.

For analysis of amino acids and TCA cycle intermediates, the samples from the HILIC analysis samples were dried and reconstituted in the same volume of aqueous 10 mM ammonium acetate before orthogonal mixed-mode analysis using an ACE Excel C18-PFP column (150 mm by 1 mm, 2.0 μm; Hichrom). Mobile phase A consisted of water with 0.1% formic acid, and mobile phase B was acetonitrile with 0.1% formic acid. For gradient elution, mobile phase B was held at 0% for 1.6 min, followed by a linear gradient to 30% B over 4.0 min, a further increase to 90% over 1 min and a hold at 90% B for 1 min with re-equilibration for 1.5 min, giving a total run time of 6.5 min. The flow rate was 0.5 ml/min, and the injection volume was 3.5 μl. The needle wash used was 1:1 water:acetonitrile. Source parameters used for the Q Exactive Plus were a vaporizer temperature of 400°C; ion transfer tube temperature of 300°C; an ion spray voltage of 3.5 kV (2.5 kV for negative ion mode); and a sheath gas, auxiliary gas, and sweep gas of 55, 15, and 3 arbitrary units, respectively, with an S-lens radio frequency of 60%. A full scan of 60 to 900 mass/charge ratio (*m*/*z*) was used at a resolution of 70,000 parts per million, where positive and negative ion mode assays were run separately to maximize data points across a peak at the chosen resolution.

### LC-MS data processing

Data were acquired, processed, and integrated using Xcalibur (version 4.1; Thermo Fisher Scientific). Compound retention times were validated against known external standards. Data were collated using Excel (Microsoft, Windows 10). To obtain normalized peak areas, peak areas were corrected for the total cell number of the sample. All data involving ^13^C were corrected for the natural abundance of this isotope.

### Preparation of lentivirus and transduction

In six-well plates, 5 × 10^5^ HEK293T cells were plated 1 day before infection. Lentivirus was produced by transfection of HEK293T cells using FuGENE (Promega), according to standard procedures, using 2 μg of DNA [DNA was mixed in a 3:2:4 ratio of the relevant expression plasmid, pCMV-dR8.91 (gag/pol), and pMD.G (VSVG)] per well. Two days after transfection, supernatants were collected and passed through a 0.45-μm filter and was aliquoted in appropriate volumes and stored at −80°C.

For the screens, a multiplicity of infection (MOI) of <0.3 was achieved, which was established by titration of thawed virus supernatant that was added to a six-well plate containing 2 × 10^6^ HAP1 cells. After the virus was added, the plate was centrifuged for 1 hour at maximum speed at 37°C.

### CRISPR-Cas9 sgRNA pooled libraries

The Stanford library (Addgene, #101926-101934) was a gift from M. Bassik. The Whitehead knockout library (Addgene, #1000000095) was a gift from D. Sabatini and E. Lander.

### CRISPR-Cas9 genetic screening

HAP1 *Cas9* cells were generated by lentiviral transduction of lentiCas9-Blast (gift from F. Zhang; Addgene, #52962) and selected for Cas9 expression using blasticidin (20 μg/ml) for at least 7 days. Single-cell clones were obtained as described above. Clonal HAP1 Cas9 cells were then transduced with the sgRNA libraries at an MOI of <0.3. After 48 hours, cells were selected for 3 days with puromycin (3.5 μg/ml; Gibco). At least 250-fold sgRNA coverage was maintained throughout the screening procedure.

Genomic DNA isolation was performed using a Gentra Puregene Core kit (QIAGEN). Amplification of the lentiviral inserts containing the sgRNA sequences was performed with a nested polymerase chain reaction (PCR; primer sequences can be found in table S2) to create sequencing libraries. Next-generation sequencing was performed on a HiSeq-4000 platform (Illumina) according to standard procedures.

Sequence analysis was performed as follows: The vector sequence (GTT TAA GAG CTA AGC TGG AAA CAG CAT AGC AA) was clipped off (Stanford library), or just the first 20 base pairs were kept (Whitehead library) of each read using Cutadapt (version 3.7) (*34*). The resulting sequences were then aligned against the relevant sgRNA library using Bowtie2 (version 2.4.3) while allowing no mismatches (*35*). Each sample had >85% uniquely mapping reads. Unmapped/multimapping reads were discarded using Samtools (*36*). To generate statistical scores for either depletion or enrichment of sgRNAs between the conditions, MAGeCK (0.5.9.4) was used (*37*). These scores can be found in data files S1 to S3.

### Gene ontology analysis

Gene ontology analysis was performed with DAVID (*38*) and GOplot (*39*) using standard settings.

### Flow cytometry

To quantify apoptosis, cells were grown in medium with or without HT for 7 days and stained with CellEvent Caspase-3/7 Green Detection Reagent (Thermo Fisher Scientific) according to the manufacturer’s recommendations. Camptothecin (30 μM) was used as a positive control to induce apoptosis. Mitochondrial content was quantified by growing cells in medium with or without HT for 7 days and staining them with MitoTracker Green (Thermo Fisher Scientific) according to the manufacturer’s recommendations. Cell cycle profiles were obtained from strains carrying the F3C cell cycle reporter (described above) by growing cells in medium with or without HT for the indicated time points. On the day of analysis, cells were harvested by trypsinization.

All samples were analyzed with an LSRFortessa Cell Analyzer (BD Biosciences). Gating strategies can be found in fig. S7.

### Quantitative PCR

HAP1 cells were plated without HT for 7 days (each condition in triplicate), after which RNA was extracted from 10^6^ cells using a PureLink RNA Mini Kit (Thermo Fisher Scientific) according to standard procedures. From 1 μg of total RNA, cDNA was synthesized using ProtoScript II reverse transcriptase (NEB) and oligo dTs (Thermo Fisher Scientific) according to the manufacturer’s protocol. cDNA was then amplified with the Power SYBR Green Master Mix (Thermo Fisher Scientific) and analyzed using a ABI7900HT Real-Time PCR system (Thermo Fisher Scientific). Transcript levels were normalized to the expression of the housekeeping gene β-actin. Primer sequences can be found in table S3.

### Mitochondrial bioenergetics assays

HAP1 cells were maintained in medium without HT for 7 days before seeding at 10^5^ cells per well of a Seahorse XFe96 Analyzer plate (Agilent). After 3 to 4 hours at 37°C and 5% CO_2_, medium was replaced with serum-free Seahorse XF DMEM (Agilent) containing 1 mM pyruvate, 2 mM glutamine, and 25 mM glucose (all Agilent) for 45 min in a non-CO_2_ incubator at 37°C. These cells were then assayed on a Seahorse XFe96 Analyzer (Agilent) according to the manufacturer’s Mito Stress Test protocol. The concentration used for all the inhibitors was 1 μM. Mitochondrial bioenergetics assays were also performed with HAP1 cells in the presence of HT or formate (400 μM) and performed as described above.

Basal oxygen consumption rate (OCR) was also measured using a Resipher real-time cell analyzer (Baker): HAP1 cells were seeded at 4 × 10^5^ cells per well in a 96-well plate (Falcon) in the presence of HT. After 3 hours, OCR monitoring started by placing the device on the cells for 3 hours. Next, the medium was replaced by medium without HT, and OCR was monitored for another 10 hours.

To test the effect of metformin on OCR flux in HAP1 WT cells (+HT/) using a Seahorse XFe96 Analyzer, 4 × 10^4^ cells were plated in a Seahorse XFe96 Analyzer plate ±metformin (2.5 mM). After 24 hours, the experiment was performed as described above, except that cells that were pretreated with metformin were also exposed to metformin throughout the assay.

The effect of metformin on HAP1 WT cells was also analyzed using a Resipher real-time cell analyzer: A total of 8 × 10^5^ cells were seeded in a 96-well plate (Falcon) in the presence of HT and ±metformin (2.5 mM) and allowed to attach for 6 hours. Then, antimycin A/rotenone (both at 1 μM) was added as a control to suppress oxygen consumption, and OCR flux was monitored continuously.

To test the effect of formaldehyde on OCR flux in HAP1 WT and ∆*S1/2* strains, cells were grown without HT and ±formaldehyde (15 μM) for 3 days. Then, the cells were plated in a Seahorse XFe96 Analyzer plate in the same condition. After 6 hours, the mitochondrial bioenergetics assay was performed as above, except that cells that were pretreated with formaldehyde were also exposed to formaldehyde throughout the assay.

### Extraction of polar metabolites from [^13^C]algae as internal standard for GC-MS

Polar metabolites were extracted from 100 mg of ^13^C algal lyophilized cells (*Synechococcus* sp.; Sigma-Aldrich) in a conical tube using a double Folch extraction method (*40*): On milliliters of 2:1 chloroform:methanol was added to the sample and subsequently vortexed and sonicated as described above. Next, 400 μl of H_2_O was added, along with a metal bead (QIAGEN), and the sample was placed in a tissue lyser (SLS) according to the manufacturer’s recommendations. Afterward, the sample was centrifuged at maximum speed for 10 min at 4°C. The upper, aqueous phase containing, among others, polar metabolites was then transferred to a new conical tube, and the extraction was repeated. Last, the aqueous phase was dried under nitrogen as described above, and the dried sample was reconstituted in 240 μl of 80% methanol, including 1.7 μg of norvaline/ml and stored at −20°C. All solvents used were of LC-MS–grade purity.

### Sample preparation for GC-MS of polar metabolites

HAP1 cells were seeded in six-well plates (WT, 2 × 10^5^ cells per well; ∆*S1/2* ∆*H3*, 10^6^ cells per well; and others, 2 × 10^6^ cells per well) without HT. After 7 days, metabolites were extracted as follows: Cells were quickly washed with ice-cold saline, and 600 μl of 80% methanol, including 1.7 μg of norvaline/ml and 10 μl of internal standard/ml (described above), was added. Cells were scraped in this extraction buffer and then transferred to a 1.5-ml tube. Next, samples were vortexed for 10 min at 4°C and centrifuged for 10 min at maximum speed at 4°C. Supernatants were transferred to a new 1.5-ml tube and dried under nitrogen using a MULTIVAP nitrogen evaporator (Organomation). Samples were stored at −80°C.

*tert*-Butyldimethylsilyl derivatives of amino acids and TCA metabolites were prepared as follows: Sixteen microliters of 2% methoxyamine–hydrogen chloride in pyridine (MOX reagent; Thermo Fisher Scientific) was added to the dried samples and incubated for 1 hour at 37°C. Next, 20 μl of *N*-*tert*-butyldimethylsilyl-*N*-methyltrifluoroacetamide with 1% *tert*-butyldimethylchlorosilane (Sigma-Aldrich) was added and incubated for 30 min at 60°C. Last, samples were centrifuged at maximum speed for 5 min, and 20 μl of the derivatized sample was transferred to a glass GC-MS vial (Agilent). All samples were subjected to GC-MS within 1 week after derivatization. All solvents used were of LC-MS–grade purity.

### GC-MS of polar metabolites

GC-MS analysis was performed as previously described (*41*–*43*) with modifications: Derivatized samples were analyzed by GC-MS using a DB-35MS column (Agilent Technologies) with an Agilent 7890B gas chromatograph that was coupled to an Agilent 5997B mass spectrometer. Helium was used as a carrier gas at a flow rate of 1.2 ml/min. One microliter of sample was injected in splitless mode at 270°C. The GC oven was held at 100°C for 1 min, after which it increased to 105°C at 2.5°C/min and held for 2 min, then increased to 250°C at 3.5°C/min, and lastly increased to 320°C at 20°C/min and held for 5 min. For MS, electron impact ionization at 70 eV was used. The source and detector were kept at 230° and 150°C, respectively. The detector was used in normal scanning mode, and the scanned ion range was 100 to 650 *m*/*z*.

### GC-MS data processing

Mass isotopomer distributions were determined by integrating appropriate ion fragments for each metabolite using MATLAB-based Metran software (*44*) that corrects for natural abundance using previously described methods (*45*). Metabolites were quantitated relative to their corresponding ^13^C-labeled internal standard, corrected for total cell numbers, and then expressed as fold changes relative to control. All experiments were conducted in triplicate.

### Immunoblotting

HAP1 protein extracts were prepared as follows: A total of 10^7^ cells were washed in ice-cold PBS and transferred to a 1.5-ml tube. Cell pellets were lysed in ice-cold 200-μl radioimmunoprecipitation assay buffer (Thermo Fisher Scientific) containing 1× protease inhibitors (cOmplete Protease Inhibitor Cocktail tablets, Roche) and 0.2 μl of DENARASE (c-LEcta) and left on ice for 30 min. For sample reduction, 1× NuPAGE LDS Sample Buffer (Thermo Fisher Scientific), containing 5% 2-mercaptoethanol (Sigma-Aldrich), was added, and the tube was placed at 95°C for 5 min. Before immunoblotting, samples were stored at −20°C.

For immunoblotting of HAP1 samples, 15 μl of the extracts described above were loaded on a 4 to 12% NuPAGE bis-tris gel and run according to standard methods. Samples were blotted to a 0.45-μm nitrocellulose membrane. All antibodies that have been used can be found in table S4.

### RNA sequencing analysis

Raw sequencing data were obtained from GSE127973. First adapter sequences were quality-trimmed using Trim Galore 0.6.10 using standard settings (DOI 10.5281/zenodo.7598955). Transcript abundance was quantified using Salmon against protein coding transcript sequences (GENCODE v35) using standard settings (*46*), and differential gene expression analysis was performed using DESeq2 using standard settings (*47*).

### Visualization of genetic screen hits in complex I structure

The mammalian (*Bos taurus*) complex I structure (5LDW, Protein Data Bank) (*18*) was loaded into PyMOL (Schrödinger), and all complex I subunits found in the genetic screen (chains a, g, F, G, k, m, n, O, S, and X) were either marked red using the command “color br8, chain a” for core subunits or marked blue using the command “color skyblue for supernumerary subunits, chain a,” except for the two assembly subunits tht are not present in the structure. Other settings used with PyMOL were “set spec_refl = 1.5,” “set spec_power = 200,” “set ray_trace_fog = 0,” “set depth_cue = 0,” “color silver,” “bg_color white,” and “show surface.”

### Statistical analyses

Statistical analyses were performed with GraphPad Prism 9 software. We determined the statistical significance of differences between two groups using the unpaired two-tailed Student’s *t* test and among more than two groups using one- or two-way analysis of variance (ANOVA) analysis with Tukey’s multiple comparisons test. Statistical analysis on contingency tables was performed using pairwise chi-square tests of average distributions.

## References

[1] J. A. Swenberg, B. C. Moeller, K. Lu, J. E. Rager, R. C. Fry, T. B. Starr, Formaldehyde carcinogenicity research: 30 years and counting for mode of action, epidemiology, and cancer risk assessment. Toxicol. Pathol. 41, 181–189 (2013). 23160431 10.1177/0192623312466459 PMC3893912

[2] F. A. Dingler, M. Wang, A. Mu, C. L. Millington, N. Oberbeck, S. Watcham, L. B. Pontel, A. N. Kamimae-Lanning, F. Langevin, C. Nadler, R. L. Cordell, P. S. Monks, R. Yu, N. K. Wilson, A. Hira, K. Yoshida, M. Mori, Y. Okamoto, Y. Okuno, H. Muramatsu, Y. Shiraishi, M. Kobayashi, T. Moriguchi, T. Osumi, M. Kato, S. Miyano, E. Ito, S. Kojima, H. Yabe, M. Yabe, K. Matsuo, S. Ogawa, B. Göttgens, M. R. G. Hodskinson, M. Takata, K. J. Patel, Two aldehyde clearance systems are essential to prevent lethal formaldehyde accumulation in mice and humans. Mol. Cell 80, 996–1012.e9 (2020). 33147438 10.1016/j.molcel.2020.10.012 PMC7758861

[3] L. Uotila, M. Koivusalo, Formaldehyde dehydrogenase from human liver. J. Biol. Chem. 249, 7653–7663 (1974). 4373474

[4] L. B. Pontel, I. V. Rosado, G. Burgos-Barragan, J. I. Garaycoechea, R. Yu, M. J. Arends, G. Chandrasekaran, V. Broecker, W. Wei, L. Liu, J. A. Swenberg, G. P. Crossan, K. J. Patel, Endogenous formaldehyde is a hematopoietic stem cell genotoxin and metabolic carcinogen. Mol. Cell 60, 177–188 (2015). 26412304 10.1016/j.molcel.2015.08.020 PMC4595711

[5] L. Mulderrig, J. I. Garaycoechea, Z. K. Tuong, C. L. Millington, F. A. Dingler, J. R. Ferdinand, L. Gaul, J. A. Tadross, M. J. Arends, S. O’Rahilly, G. P. Crossan, M. R. Clatworthy, K. J. Patel, Aldehyde-driven transcriptional stress triggers an anorexic DNA damage response. Nature 600, 158–163 (2021). 34819667 10.1038/s41586-021-04133-7

[6] G. Burgos-Barragan, N. Wit, J. Meiser, F. A. Dingler, M. Pietzke, L. Mulderrig, L. B. Pontel, I. V. Rosado, T. F. Brewer, R. L. Cordell, P. S. Monks, C. J. Chang, A. Vazquez, K. J. Patel, Mammals divert endogenous genotoxic formaldehyde into one-carbon metabolism. Nature 548, 549–554 (2017). 28813411 10.1038/nature23481 PMC5714256

[7] R. J. Hopkinson, R. B. Hamed, N. R. Rose, T. D. Claridge, C. J. Schofield, Monitoring the activity of 2-oxoglutarate dependent histone demethylases by NMR spectroscopy: Direct observation of formaldehyde. Chembiochem 11, 506–510 (2010). 20095001 10.1002/cbic.200900713

[8] G. S. Ducker, J. D. Rabinowitz, One-carbon metabolism in health and disease. Cell Metab. 25, 27–42 (2017). 27641100 10.1016/j.cmet.2016.08.009 PMC5353360

[9] D. W. Morgens, M. Wainberg, E. A. Boyle, O. Ursu, C. L. Araya, C. K. Tsui, M. S. Haney, G. T. Hess, K. Han, E. E. Jeng, A. Li, M. P. Snyder, W. J. Greenleaf, A. Kundaje, M. C. Bassik, Genome-scale measurement of off-target activity using Cas9 toxicity in high-throughput screens. Nat. Commun. 8, 15178 (2017). 28474669 10.1038/ncomms15178 PMC5424143

[10] R. J. Park, T. Wang, D. Koundakjian, J. F. Hultquist, P. Lamothe-Molina, B. Monel, K. Schumann, H. Yu, K. M. Krupzcak, W. Garcia-Beltran, A. Piechocka-Trocha, N. J. Krogan, A. Marson, D. M. Sabatini, E. S. Lander, N. Hacohen, B. D. Walker, A genome-wide CRISPR screen identifies a restricted set of HIV host dependency factors. Nat. Genet. 49, 193–203 (2017). 27992415 10.1038/ng.3741 PMC5511375

[11] M. J. Emmett, M. A. Lazar, Integrative regulation of physiology by histone deacetylase 3. Nat. Rev. Mol. Cell Biol. 20, 102–115 (2019). 30390028 10.1038/s41580-018-0076-0 PMC6347506

[12] A. A. Duarte, E. Gogola, N. Sachs, M. Barazas, S. Annunziato, J. R de Ruiter, A. Velds, S. Blatter, J. M. Houthuijzen, M. van de Ven, H. Clevers, P. Borst, J. Jonkers, S. Rottenberg, BRCA-deficient mouse mammary tumor organoids to study cancer-drug resistance. Nat. Methods 15, 134–140 (2018). 29256493 10.1038/nmeth.4535

[13] M. Malvaez, S. C. McQuown, G. A. Rogge, M. Astarabadi, V. Jacques, S. Carreiro, J. R. Rusche, M. A. Wood, HDAC3-selective inhibitor enhances extinction of cocaine-seeking behavior in a persistent manner. Proc. Natl. Acad. Sci. U.S.A. 110, 2647–2652 (2013). 23297220 10.1073/pnas.1213364110 PMC3574934

[14] V. V. Ogryzko, R. L. Schiltz, V. Russanova, B. H. Howard, Y. Nakatani, The transcriptional coactivators p300 and CBP are histone acetyltransferases. Cell 87, 953–959 (1996). 8945521 10.1016/s0092-8674(00)82001-2

[15] T. Shimazu, S. Horinouchi, M. Yoshida, Multiple histone deacetylases and the CREB-binding protein regulate pre-mRNA 3′-end processing. J. Biol. Chem. 282, 4470–4478 (2007). 17172643 10.1074/jbc.M609745200

[16] L. Chen, W. Fischle, E. Verdin, W. C. Greene, Duration of nuclear NF-kappaB action regulated by reversible acetylation. Science 293, 1653–1657 (2001). 11533489 10.1126/science.1062374

[17] J. Hirst, Mitochondrial complex I. Annu. Rev. Biochem. 82, 551–575 (2013). 23527692 10.1146/annurev-biochem-070511-103700

[18] J. Zhu, K. R. Vinothkumar, J. Hirst, Structure of mammalian respiratory complex I. Nature 536, 354–358 (2016). 27509854 10.1038/nature19095 PMC5027920

[19] W. W. Wheaton, S. E. Weinberg, R. B. Hamanaka, S. Soberanes, L. B. Sullivan, E. Anso, A. Glasauer, E. Dufour, G. M. Mutlu, G. R. S. Budigner, N. S. Chandel, Metformin inhibits mitochondrial complex I of cancer cells to reduce tumorigenesis. eLife 3, e02242 (2014). 24843020 10.7554/eLife.02242 PMC4017650

[20] X. Liang, K. M. Giacomini, Transporters involved in metformin pharmacokinetics and treatment response. J. Pharm. Sci. 106, 2245–2250 (2017). 28495567 10.1016/j.xphs.2017.04.078

[21] M. J. Emmett, H. W. Lim, J. Jager, H. J. Richter, M. Adlanmerini, L. C. Peed, E. R. Briggs, D. J. Steger, T. Ma, C. A. Sims, J. A. Baur, L. Pei, K. J. Won, P. Seale, Z. Gerhart-Hines, M. A. Lazar, Histone deacetylase 3 prepares brown adipose tissue for acute thermogenic challenge. Nature 546, 544–548 (2017). 28614293 10.1038/nature22819 PMC5826652

[22] I. Martinez-Reyes, N. S. Chandel, Mitochondrial TCA cycle metabolites control physiology and disease. Nat. Commun. 11, 102 (2020). 31900386 10.1038/s41467-019-13668-3 PMC6941980

[23] D. Lorendeau, G. Rinaldi, R. Boon, P. Spincemaille, K. Metzger, C. Jäger, S. Christen, X. Dong, S. Kuenen, K. Voordeckers, P. Verstreken, D. Cassiman, P. Vermeersch, C. Verfaillie, K. Hiller, S. M. Fendt, Dual loss of succinate dehydrogenase (SDH) and complex I activity is necessary to recapitulate the metabolic phenotype of SDH mutant tumors. Metab. Eng. 43, 187–197 (2017). 27847310 10.1016/j.ymben.2016.11.005

[24] G. S. Ducker, L. Chen, R. J. Morscher, J. M. Ghergurovich, M. Esposito, X. Teng, Y. Kang, J. D. Rabinowitz, Reversal of cytosolic one-carbon flux compensates for loss of the mitochondrial folate pathway. Cell Metab. 23, 1140–1153 (2016). 27211901 10.1016/j.cmet.2016.04.016 PMC4909566

[25] J. W. Rensvold, E. Shishkova, Y. Sverchkov, I. J. Miller, A. Cetinkaya, A. Pyle, M. Manicki, D. R. Brademan, Y. Alanay, J. Raiman, A. Jochem, P. D. Hutchins, S. R. Peters, V. Linke, K. A. Overmyer, A. Z. Salome, A. S. Hebert, C. E. Vincent, N. W. Kwiecien, M. J. P. Rush, M. S. Westphall, M. Craven, N. A. Akarsu, R. W. Taylor, J. J. Coon, D. J. Pagliarini, Defining mitochondrial protein functions through deep multiomic profiling. Nature 606, 382–388 (2022). 35614220 10.1038/s41586-022-04765-3 PMC9310563

[26] L. Jiang, A. A. Shestov, P. Swain, C. Yang, S. J. Parker, Q. A. Wang, L. S. Terada, N. D. Adams, M. T. McCabe, B. Pietrak, S. Schmidt, C. M. Metallo, B. P. Dranka, B. Schwartz, R. J. DeBerardinis, Reductive carboxylation supports redox homeostasis during anchorage-independent growth. Nature 532, 255–258 (2016). 27049945 10.1038/nature17393 PMC4860952

[27] Q. S. Zhang, W. Tang, M. Deater, N. Phan, A. N. Marcogliese, H. Li, M. al-Dhalimy, A. Major, S. Olson, R. J. Monnat Jr., M. Grompe, Metformin improves defective hematopoiesis and delays tumor formation in Fanconi anemia mice. Blood 128, 2774–2784 (2016). 27756748 10.1182/blood-2015-11-683490 PMC5159699

[28] Y. Oka, M. Hamada, Y. Nakazawa, H. Muramatsu, Y. Okuno, K. Higasa, M. Shimada, H. Takeshima, K. Hanada, T. Hirano, T. Kawakita, H. Sakaguchi, T. Ichimura, S. Ozono, K. Yuge, Y. Watanabe, Y. Kotani, M. Yamane, Y. Kasugai, M. Tanaka, T. Suganami, S. Nakada, N. Mitsutake, Y. Hara, K. Kato, S. Mizuno, N. Miyake, Y. Kawai, K. Tokunaga, M. Nagasaki, S. Kito, K. Isoyama, M. Onodera, H. Kaneko, N. Matsumoto, F. Matsuda, K. Matsuo, Y. Takahashi, T. Mashimo, S. Kojima, T. Ogi, Digenic mutations in *ALDH2* and *ADH5* impair formaldehyde clearance and cause a multisystem disorder, AMeD syndrome. Sci. Adv. 6, eabd7197 (2020). 33355142 10.1126/sciadv.abd7197 PMC11206199

[29] A. Rodriguez-Nuevo, A. Torres-Sanchez, J. M. Duran, C. De Guirior, M. A. Martínez-Zamora, E. Böke, Oocytes maintain ROS-free mitochondrial metabolism by suppressing complex I. Nature 607, 756–761 (2022). 35859172 10.1038/s41586-022-04979-5 PMC9329100

[30] S. Ravera, D. Vaccaro, P. Cuccarolo, M. Columbaro, C. Capanni, M. Bartolucci, I. Panfoli, A. Morelli, C. Dufour, E. Cappelli, P. Degan, Mitochondrial respiratory chain complex I defects in Fanconi anemia complementation group A. Biochimie 95, 1828–1837 (2013). 23791750 10.1016/j.biochi.2013.06.006

[31] H. Arakawa, D. Lodygin, J. M. Buerstedde, Mutant loxP vectors for selectable marker recycle and conditional knock-outs. BMC Biotechnol. 1, 7 (2001). 11591226 10.1186/1472-6750-1-7 PMC57747

[32] G. S. Ducker, J. M. Ghergurovich, N. Mainolfi, V. Suri, S. K. Jeong, S. Hsin-Jung Li, A. Friedman, M. G. Manfredi, Z. Gitai, H. Kim, J. D. Rabinowitz, Human SHMT inhibitors reveal defective glycine import as a targetable metabolic vulnerability of diffuse large B-cell lymphoma. Proc. Natl. Acad. Sci. U.S.A. 114, 11404–11409 (2017). 29073064 10.1073/pnas.1706617114 PMC5664509

[33] M. Z. Cader, R. P. de Almeida Rodrigues, J. A. West, G. W. Sewell, M. N. Md-Ibrahim, S. Reikine, G. Sirago, L. W. Unger, A. B. Iglesias-Romero, K. Ramshorn, L. M. Haag, S. Saveljeva, J. F. Ebel, P. Rosenstiel, N. C. Kaneider, J. C. Lee, T. D. Lawley, A. Bradley, G. Dougan, Y. Modis, J. L. Griffin, A. Kaser, FAMIN is a multifunctional purine enzyme enabling the purine nucleotide cycle. Cell 180, 278–295.e23 (2020). 31978345 10.1016/j.cell.2019.12.017 PMC6978800

[34] M. Martin, CUTADAPT removes adapter sequences from high-throughput sequencing reads. EMBnet.journal 17, 10–12 (2011).

[35] B. Langmead, S. L. Salzberg, Fast gapped-read alignment with Bowtie 2. Nat. Methods 9, 357–359 (2012). 22388286 10.1038/nmeth.1923 PMC3322381

[36] H. Li, B. Handsaker, A. Wysoker, T. Fennell, J. Ruan, N. Homer, G. Marth, G. Abecasis, R. Durbin; 1000 Genome Project Data Processing Subgroup, The sequence alignment/map format and SAMtools. Bioinformatics 25, 2078–2079 (2009). 19505943 10.1093/bioinformatics/btp352 PMC2723002

[37] W. Li, H. Xu, T. Xiao, L. Cong, M. I. Love, F. Zhang, R. A. Irizarry, J. S. Liu, M. Brown, X. S. Liu, MAGeCK enables robust identification of essential genes from genome-scale CRISPR/Cas9 knockout screens. Genome Biol. 15, 554 (2014). 25476604 10.1186/s13059-014-0554-4 PMC4290824

[38] D. W. Huang, B. T. Sherman, Q. Tan, J. R. Collins, W. G. Alvord, J. Roayaei, R. Stephens, M. W. Baseler, H. C. Lane, R. A. Lempicki, The DAVID gene functional classification tool: A novel biological module-centric algorithm to functionally analyze large gene lists. Genome Biol. 8, R183 (2007). 17784955 10.1186/gb-2007-8-9-r183 PMC2375021

[39] W. Walter, F. Sanchez-Cabo, M. Ricote, GOplot: An R package for visually combining expression data with functional analysis. Bioinformatics 31, 2912–2914 (2015). 25964631 10.1093/bioinformatics/btv300

[40] J. Folch, M. Lees, G. H. Sloane Stanley, A simple method for the isolation and purification of total lipides from animal tissues. J. Biol. Chem. 226, 497–509 (1957). 13428781

[41] M. R. Antoniewicz, J. K. Kelleher, G. Stephanopoulos, Accurate assessment of amino acid mass isotopomer distributions for metabolic flux analysis. Anal. Chem. 79, 7554–7559 (2007). 17822305 10.1021/ac0708893

[42] A. Muir, L. V. Danai, D. Y. Gui, C. Y. Waingarten, C. A. Lewis, M. G. Vander Heiden, Environmental cystine drives glutamine anaplerosis and sensitizes cancer cells to glutaminase inhibition. eLife 6, e27713 (2017). 28826492 10.7554/eLife.27713 PMC5589418

[43] C. A. Lewis, S. J. Parker, B. P. Fiske, D. McCloskey, D. Y. Gui, C. R. Green, N. I. Vokes, A. M. Feist, M. G. Vander Heiden, C. M. Metallo, Tracing compartmentalized NADPH metabolism in the cytosol and mitochondria of mammalian cells. Mol. Cell 55, 253–263 (2014). 24882210 10.1016/j.molcel.2014.05.008 PMC4106038

[44] J. D. Young, J. L. Walther, M. R. Antoniewicz, H. Yoo, G. Stephanopoulos, An elementary metabolite unit (EMU) based method of isotopically nonstationary flux analysis. Biotechnol. Bioeng. 99, 686–699 (2008). 17787013 10.1002/bit.21632

[45] C. A. Fernandez, C. Des Rosiers, S. F. Previs, C. F. David, H. Brunengraber, Correction of 13C mass isotopomer distributions for natural stable isotope abundance. J. Mass Spectrom. 31, 255–262 (1996). 8799277 10.1002/(SICI)1096-9888(199603)31:3<255::AID-JMS290>3.0.CO;2-3

[46] R. Patro, G. Duggal, M. I. Love, R. A. Irizarry, C. Kingsford, Salmon provides fast and bias-aware quantification of transcript expression. Nat. Methods 14, 417–419 (2017). 28263959 10.1038/nmeth.4197 PMC5600148

[47] M. I. Love, W. Huber, S. Anders, Moderated estimation of fold change and dispersion for RNA-seq data with DESeq2. Genome Biol. 15, 550 (2014). 25516281 10.1186/s13059-014-0550-8 PMC4302049

