# Supplementary material for: A histone deacetylase 3 and mitochondrial complex I axis regulates toxic formaldehyde production
Source: Sci Adv. 2023 May 17;9(20):eadg2235. doi: 10.1126/sciadv.adg2235 (PMC10191432; doi:10.1126/sciadv.adg2235)
Supplement: Supplementary file 1 — Figs. S1 to S7 Tables S1 to S4 Legends for data files S1 to S3 [file sciadv.adg2235_sm.pdf]

Science Advances
AAAS

Supplementary Materials for

A histone deacetylase 3 and mitochondrial complex I axis regulates toxic formaldehyde production

Niek Wit *et al.*

Corresponding author: James A. Nathan, jan33@cam.ac.uk; Ketan J. Patel, ketan.patel@imm.ox.ac.uk

Sci. Adv. **9**, eadg2235 (2023)
DOI: 10.1126/sciadv.adg2235

The PDF file includes:

Figs. S1 to S7
Tables S1 to S4
Legends for data files S1 to S3

Other Supplementary Material for this manuscript includes the following:

Data files S1 to S3

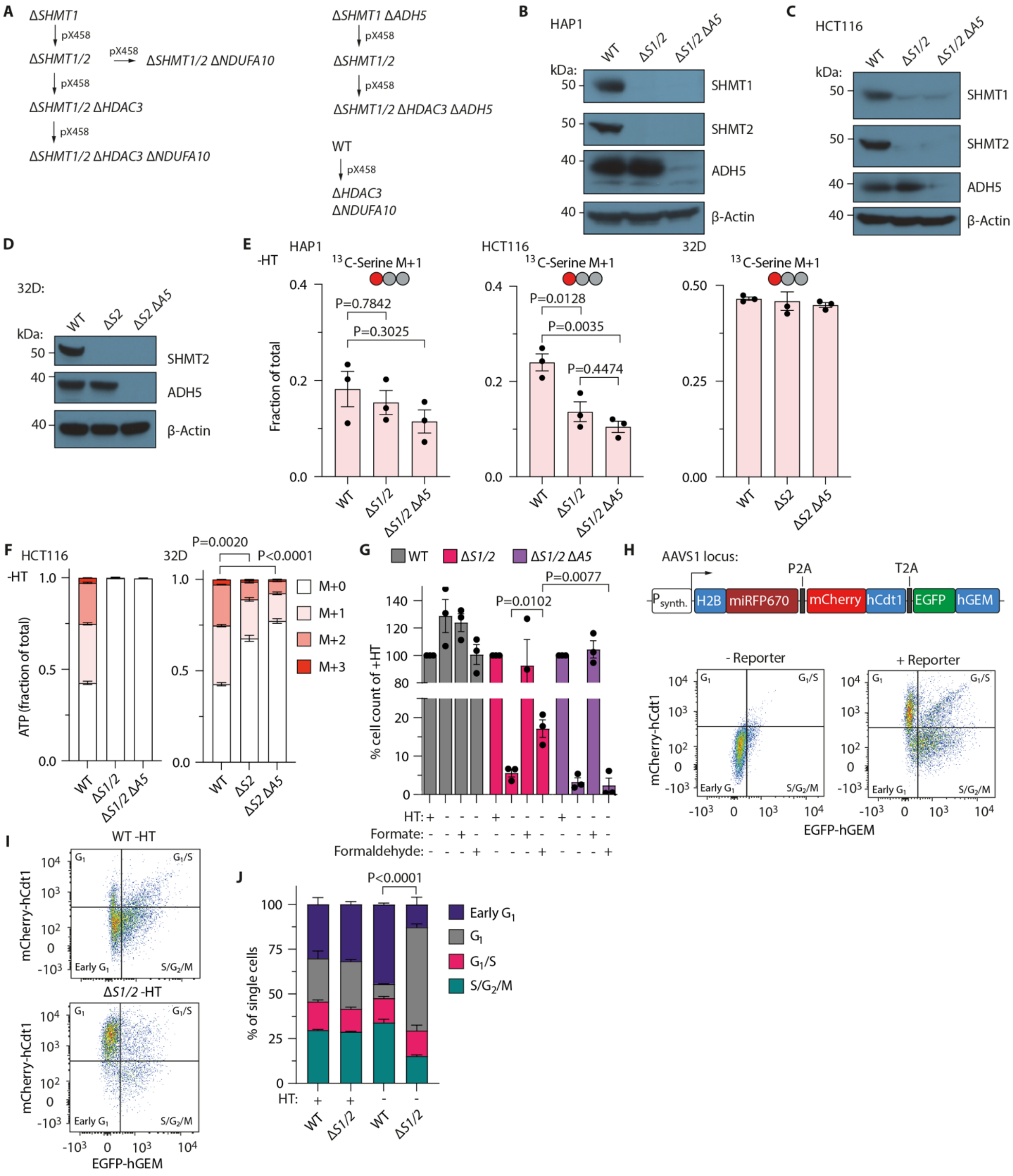

Fig. S1. (**A**) Scheme outlining the sequential strategy used to generate a panel of CRISPR-Cas9-mediated knockout cell lines in HAP1. Each genetic disruption has been generated with wild-type Streptococcus pyogenes *Cas9* (pX458). (**B**) Analysis of SHMT1, SHMT2 and ADH5 in HAP1 strains by immunoblotting. (**C**) Analysis of SHMT1, SHMT2 and ADH5 in HCT116 strains by immunoblotting. (**D**) Analysis of SHMT2 and ADH5 in 32D strains by immunoblotting. (**E**) Fraction of [^{13}C]serine M+1 in HAP1 cells fed with [3-^{13}C]serine (24 hours

-HT) (mean +/- SEM, n=3). (**F**) Fractional isotopic labelling of ATP in HCT116 and 32D cells fed [3-^{13}C]serine (mean +/- SEM, n=3) (right panel) (24 hours -HT). (**G**) Cell growth after 3 days of HAP1 strains in media with (+) or without (-) hypoxanthine (100 μM) and thymidine (16 μM) (HT), sodium formate (400 μM) or formaldehyde (50 μM) (mean +/- SEM, n=3). (**H**) Schematic of fluorescent reporter to determine cell cycle profile (top panel). Example flow cytometry data of fluorescent cell cycle reporter (bottom panels). (**I**) Representative example of flowcytometric analysis of cell cycle progression using the fluorescent reporter described in (G). (**J**) Cell cycle analysis of HAP1 strains with and without HT (mean +/- SEM, n=3) (7 days -HT). P values were determined by one-way ANOVA (E), two-way ANOVA (F) or pairwise χ^2 tests of average distributions (J).

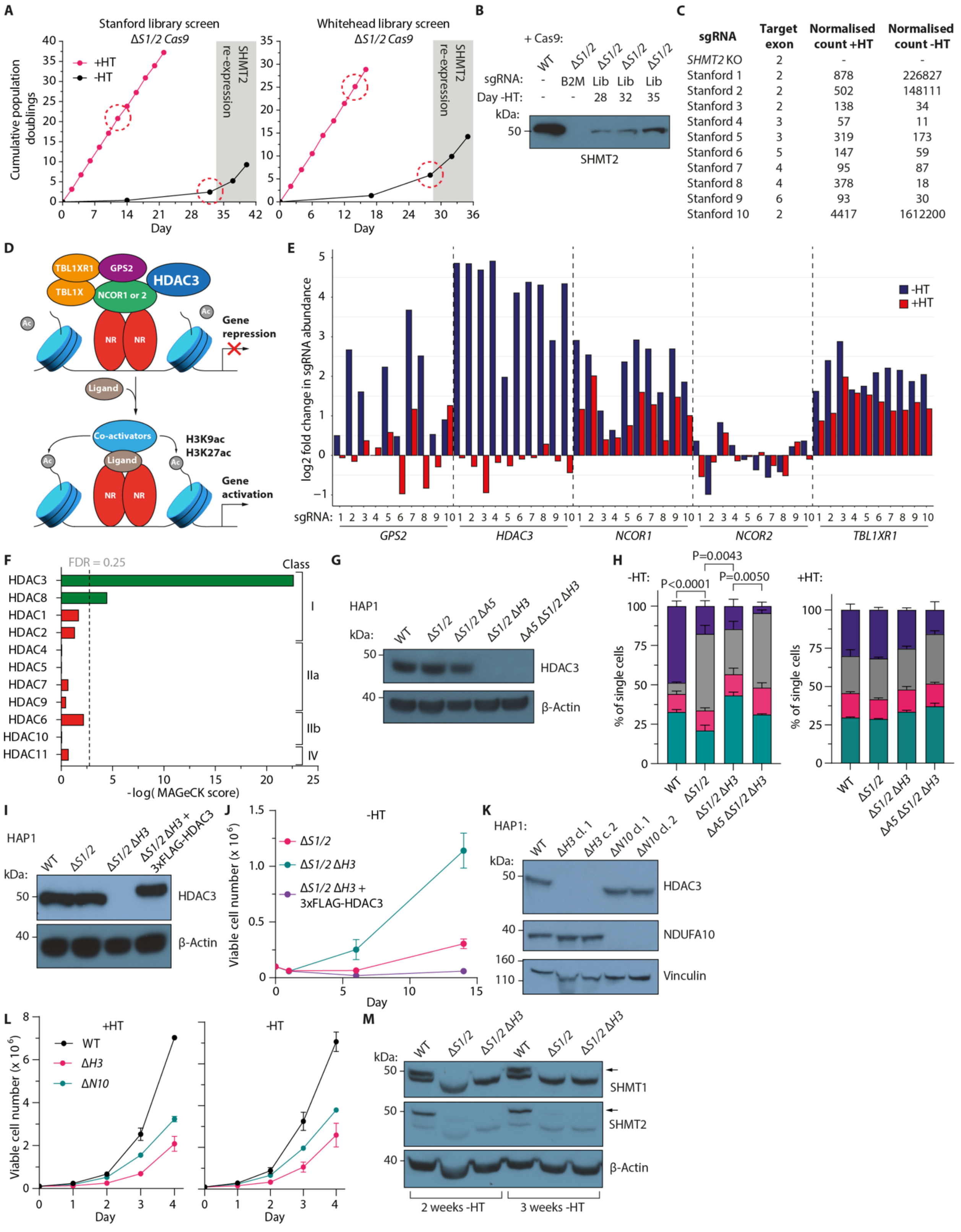

Fig. S2. (**A**) Cumulative population doublings of HAP1 Δ*S1/2 Cas9* during formaldehyde suppressor CRISPR-Cas9 screens (left panel: Stanford whole-genome sgRNA library, right

panel: Whitehead whole-genome sgRNA library. Dashed circles show which samples have been compared. (**B**) Analysis of SHMT2 in CRISPR-Cas9 screen samples or controls by immunoblotting. (**C**) Normalized counts of SHMT2 sgRNAs in growth medium with or without HT and their genomic location. (**D**) Schematic of the HDAC3 repressor complex (*11*). (**E**) Enrichment of sgRNAs targeting HDAC3 and its complex members in without HT. Values indicate log2-transformed fold change in abundance compared to plasmid. (**F**) CRISPR screen scores (MAGeCK) for all HDACs of the Stanford library screen. (**G**) Analysis of HDAC3 in HAP1 strains by immunoblotting. (**H**) Cell cycle analysis of HAP1 strains with and without HT (7 days) (mean +/- SEM, n=3). (**I**) Analysis of HDAC3 in HAP1 strains by immunoblotting. (**J**) Growth curves of HAP1 strains with (+) and without (-) HT complemented with 3xFLAG-HDAC3 (mean +/- SEM, n=3). (**K**) Analysis of HDAC3 and NDUFA10 in HAP1 strains by immunoblotting. (**L**) Growth curves of HAP1 strains with (+) and without (-) HT (mean +/- SEM, n=3). (**M**) Analysis of SHMT1, SHMT2 in HAP1 strains by immunoblotting. P values were determined by pairwise χ^2 tests of average distributions.

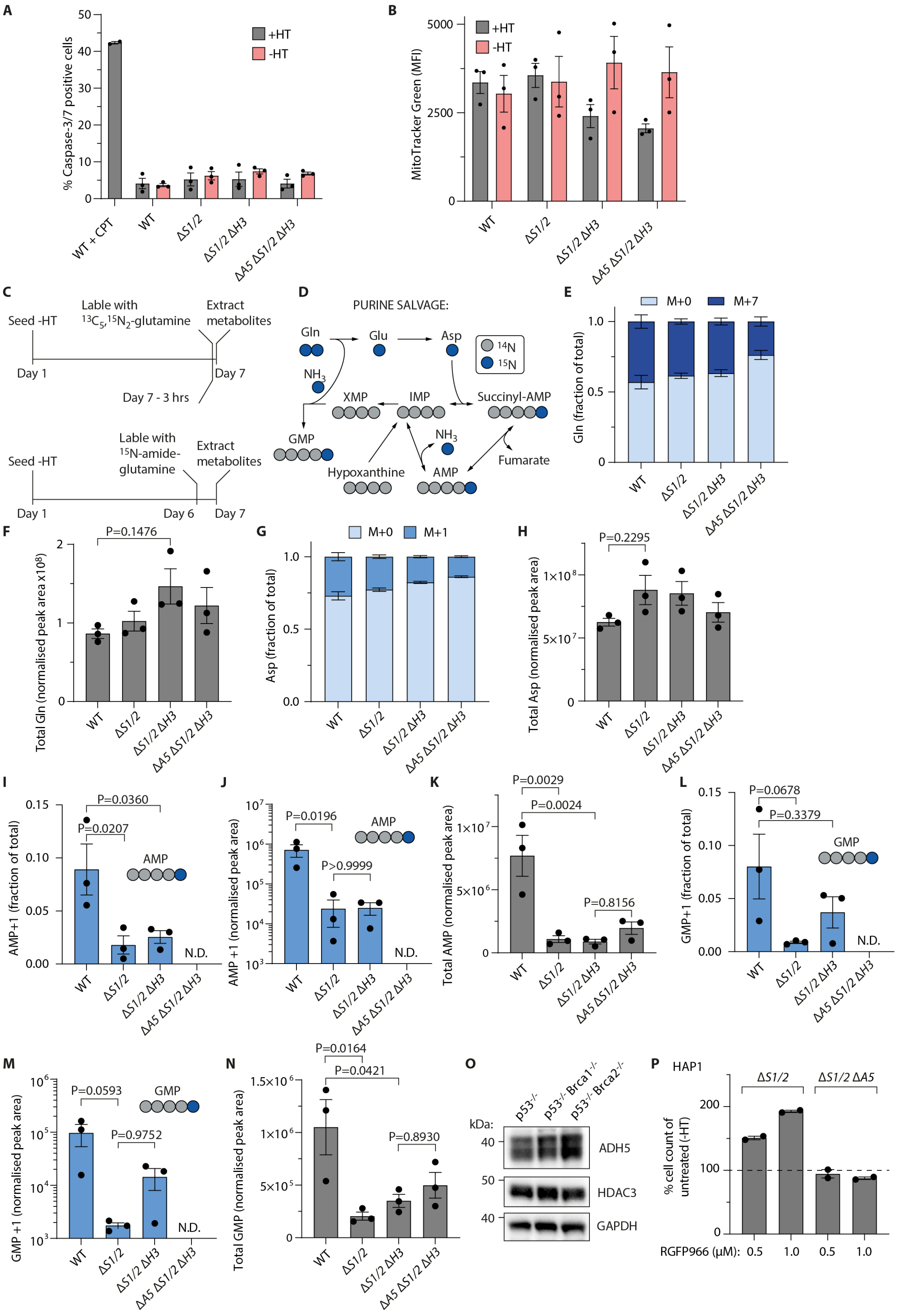

A
% Caspase-3/7 positive cells
+HT
-HT
WT + CPT
WT
ΔS1/2
ΔS1/2 ΔH3
ΔA5 ΔS1/2 ΔH3
B
MitoTracker Green (MFI)
C
Seed -HT
Lable with $^{13}C_5$,$^{15}N_2$-glutamine
Extract metabolites
Day 1
Day 7
Day 7 - 3 hrs
Lable with ^{15}N-amide-glutamine
Day 6
D
PURINE SALVAGE:
Gln
Glu
Asp
NH_3
XMP
IMP
Succinyl-AMP
GMP
Hypoxanthine
AMP
Fumarate
^{14}N
^{15}N
E
M+0
M+7
Gln (fraction of total)
F
P=0.1476
Total Gln (normalised peak area x10^8)
G
M+1
Asp (fraction of total)
H
P=0.2295
Total Asp (normalised peak area)
I
P=0.0360
P=0.0207
AMP+1 (fraction of total)
N.D.
J
P=0.0196
P>0.9999
AMP +1 (normalised peak area)
K
P=0.0029
P=0.0024
P=0.8156
Total AMP (normalised peak area)
L
P=0.0678
P=0.3379
GMP+1 (fraction of total)
M
P=0.0593
P=0.9752
GMP +1 (normalised peak area)
N
P=0.0164
P=0.0421
P=0.8930
Total GMP (normalised peak area)
O
p53$^{-/-}$
p53$^{-/-}$ Brca1$^{-/-}$
p53$^{-/-}$ Brca2$^{-/-}$
kDa:
ADH5
HDAC3
GAPDH
P
HAP1
ΔS1/2
ΔS1/2 ΔA5
% cell count of untreated (-HT)
RGFP966 (μM):

Fig. S3. (**A**) Quantification of apoptosis in HAP1 strains with or without HT (7 days) (mean +/- SEM, n=3). (**B**) Quantification of mitochondrial content in HAP1 strains with or without HT (7 days) (mean +/- SEM, n=3). (**C**) Schematics for the labelling strategy to assess purine salvage pathway activity (top panel) or de novo purine synthesis (bottom panel). (**D**) Metabolic tracing strategy to determine purine salvage activities using [$^{15}N_2$,$^{13}C_5$]glutamine. (**E**) Fractional isotopic labelling of glutamine (Gln) in HAP1 strains fed [$^{15}N_2$,$^{13}C_5$]glutamine (mean +/- SEM, n=3). (**F**) Total normalised peak areas of Gln in HAP1 strains (mean +/- SEM, n=3). (**G**) Fractional isotopic labelling of aspartate (Asp) in HAP1 strains fed [$^{15}N_2$,$^{13}C_5$]glutamine (mean +/- SEM, n=3). (**H**) Total normalised peak areas of Asp in HAP1 strains (mean +/- SEM, n=3). (**I**) Fractional labelling of ^{15}N AMP+1 in HAP1 strains (mean +/- SEM, n=3). (**J**) Normalised peak areas of ^{15}N AMP+1 in HAP1 strains (mean +/- SEM, n=3). (**K**) Total normalised peak areas of AMP in HAP1 strains (mean +/- SEM, n=3). (**L**) Fractional labelling of ^{15}N GMP+1 in HAP1 strains (mean +/- SEM, n=3). (**M**) Normalised peak areas of ^{15}N GMP+1 in HAP1 strains (mean +/- SEM, n=3). (**N**) Total normalised peak areas of GMP in HAP1 strains (mean +/- SEM, n=3). (**O**) Analysis of ADH5 and HDAC3 in tumor organoids by immunoblotting. (**P**) Cell growth -HT, with two doses of RGFP966 in HAP1 Δ*S1/2* and Δ*S1/2* Δ*A5* cells. Growth relative to untreated (day 11 -HT) (mean +/- SEM, n=2). P values were determined by one-way ANOVA.

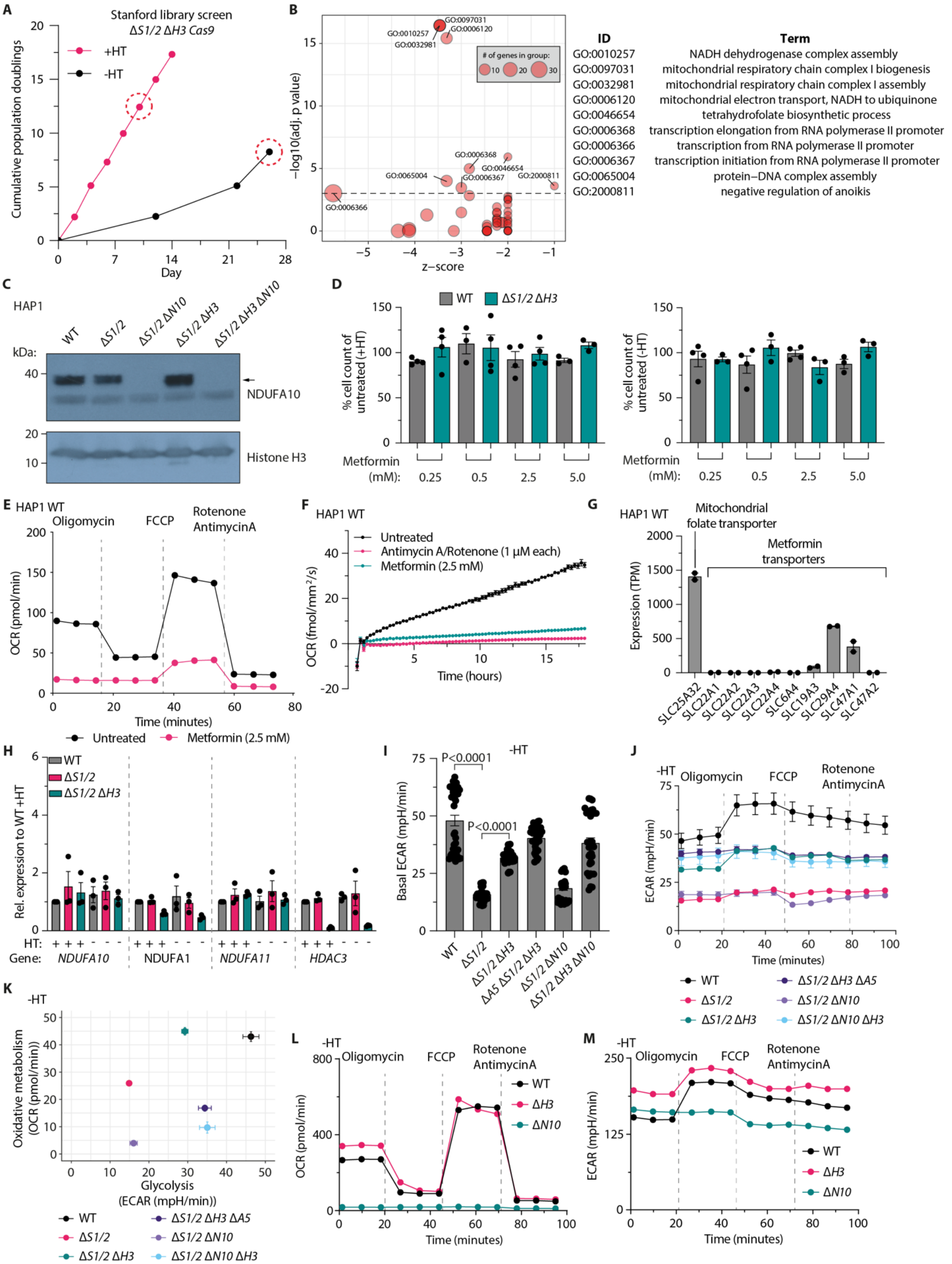

A
Stanford library screen
ΔS1/2 ΔH3 Cas9
+HT
-HT
Cumulative population doublings
Day
B
-log10(adj. p value)
z-score
# of genes in group:
10 20 30
GO:0097031
GO:0006120
GO:0010257
GO:0032981
GO:0006368
GO:0046654
GO:0065004
GO:0006367
GO:2000811
GO:0006366
ID
Term
GO:0010257 NADH dehydrogenase complex assembly
GO:0097031 mitochondrial respiratory chain complex I biogenesis
GO:0032981 mitochondrial respiratory chain complex I assembly
GO:0006120 mitochondrial electron transport, NADH to ubiquinone
GO:0046654 tetrahydrofolate biosynthetic process
GO:0006368 transcription elongation from RNA polymerase II promoter
GO:0006366 transcription from RNA polymerase II promoter
GO:0006367 transcription initiation from RNA polymerase II promoter
GO:0065004 protein–DNA complex assembly
GO:2000811 negative regulation of anoikis
C
HAP1
WT
ΔS1/2
ΔS1/2 ΔN10
ΔS1/2 ΔH3
ΔS1/2 ΔH3 ΔN10
kDa:
NDUFA10
Histone H3
D
WT
ΔS1/2 ΔH3
% cell count of untreated (+HT)
% cell count of untreated (-HT)
Metformin (mM):
0.25 0.5 2.5 5.0
E
HAP1 WT
Oligomycin
FCCP
Rotenone AntimycinA
OCR (pmol/min)
Time (minutes)
Untreated
Metformin (2.5 mM)
F
HAP1 WT
Untreated
Antimycin A/Rotenone (1 μM each)
Metformin (2.5 mM)
OCR (fmol/mm²/s)
Time (hours)
G
HAP1 WT
Mitochondrial folate transporter
Metformin transporters
Expression (TPM)
SLC25A32
SLC22A1
SLC22A2
SLC22A3
SLC22A4
SLC6A4
SLC19A3
SLC29A4
SLC47A1
SLC47A2
H
WT
ΔS1/2
ΔS1/2 ΔH3
Rel. expression to WT +HT
HT:
Gene:
NDUFA10
NDUFA1
NDUFA11
HDAC3
I
-HT
P<0.0001
P<0.0001
Basal ECAR (mpH/min)
WT
ΔS1/2
ΔS1/2 ΔH3
ΔA5 ΔS1/2 ΔH3
ΔS1/2 ΔN10
ΔS1/2 ΔH3 ΔN10
J
-HT
Oligomycin
FCCP
Rotenone AntimycinA
ECAR (mpH/min)
Time (minutes)
WT
ΔS1/2
ΔS1/2 ΔH3
ΔS1/2 ΔH3 ΔA5
ΔS1/2 ΔN10
ΔS1/2 ΔN10 ΔH3
K
-HT
Oxidative metabolism (OCR (pmol/min))
Glycolysis (ECAR (mpH/min))
WT
ΔS1/2
ΔS1/2 ΔH3
ΔS1/2 ΔH3 ΔA5
ΔS1/2 ΔN10
ΔS1/2 ΔN10 ΔH3
L
-HT
Oligomycin
FCCP
Rotenone AntimycinA
WT
ΔH3
ΔN10
OCR (pmol/min)
Time (minutes)
M
-HT
Oligomycin
FCCP
Rotenone AntimycinA
ECAR (mpH/min)
WT
ΔH3
ΔN10
Time (minutes)

Fig. S4. (**A**) Cumulative population doublings of HAP1 Δ*S1/2 ΔH3 Cas9* during formaldehyde source CRISPR-Cas9 screen with the Stanford whole-genome sgRNA library. Dashed circles show which samples have been compared. (**B**) Gene ontology analysis of formaldehyde screen results in (A). (**C**) Analysis of NDUFA10 in HAP1 strains by immunoblotting. (**D**) Cell growth +/- HT, with increasing concentrations of metformin in HAP1 WT and Δ*S1/2* Δ*H3* cells. Growth relative to no metformin (day 3:all WT and +HT Δ*S1/2* Δ*H3*; day 14 -HT Δ*S1/2* Δ*H3*) (mean +/- SEM, n=3). (**E**) Bioenergetic assays of OCR in HAP1 WT in growth medium +HT (mean +/- SEM, n>24, technical replicates, representative experiment shown) +/- 2.5 mM metformin (24 hours pre-treatment). (**F**) OCR flux measurements using a Resipher of HAP1 WT cells in +HT +/- metformin (2.5mM). (**G**) HAP1 transcript levels (bulk RNA-Seq from GSE127973) of known metformin transporters. (**H**) Quantification of transcript levels of several complex I genes (3 days -HT) (mean +/- SEM, n=3). (**I**) Basal ECAR of HAP1 strains (7 days -HT) (mean +/- SEM, n>=20, technical replicates, representative experiment shown). (**J**) Extracellular acidification rate (ECAR) of HAP1 strains (7 days -HT) (mean +/- SEM, n>=20, technical replicates, representative experiment shown). (**K**) Energy profile of HAP1 strains showing both OCR and ECAR (7 days -HT). (**L**) Bioenergetic assays of OCR in HAP1 strains in growth medium without HT (7 days) (mean +/- SEM, n>=20, technical replicates, representative experiment shown). (**M**) Bioenergetic assays of ECAR in HAP1 strains in growth medium without HT (7 days) (mean +/- SEM, n>=20, technical replicates, representative experiment shown). P values were determined by one-way ANOVA. P values were determined by one-way ANOVA.

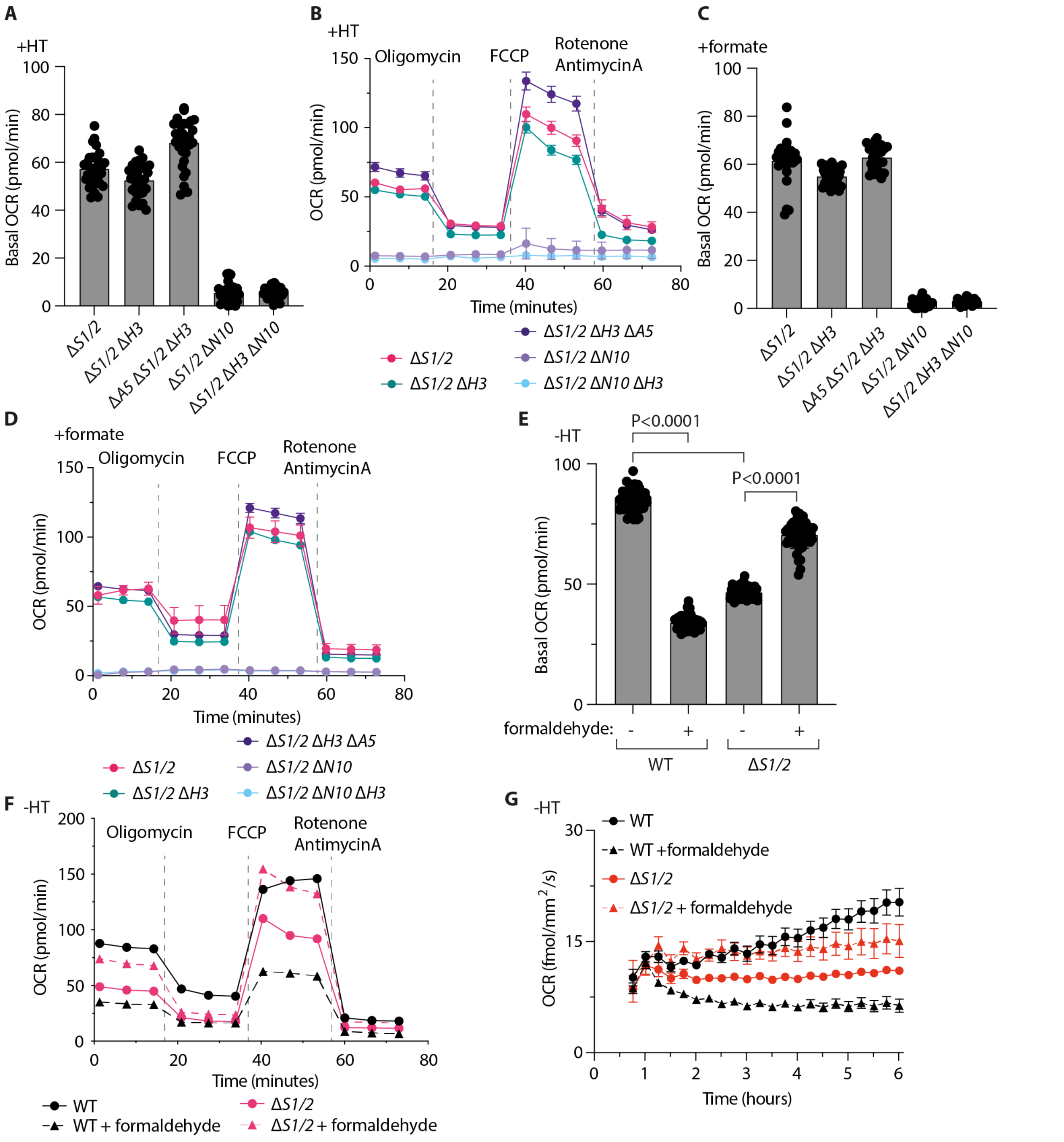

Fig. S5. (**A**) Basal oxygen consumption rate (OCR) with HT of various HAP1 genotypes obtained with a Seahorse Xfe 96 Analyzer (mean +/- SEM, n=>12, technical replicates, representative experiment shown). (**B**) Bioenergetic assays of OCR in HAP1 strains in growth medium with HT (mean +/- SEM, n=>12, technical replicates, representative experiment shown). (**C**) Basal oxygen consumption rate (OCR) with formate (400 µM) and without HT (7 days) of various HAP1 genotypes obtained with a Seahorse Xfe 96 Analyzer (mean +/- SEM, n=>12, technical replicates, representative experiment shown). (**D**) Bioenergetic assays of OCR in HAP1 strains in growth medium with formate (400 µM) and without HT (7 days) (mean +/- SEM, n=>12, technical replicates, representative experiment shown). (**E**) Basal oxygen consumption

rate (OCR) with formaldehyde (15 μM) and without HT (3 days) of HAP1 WT and Δ*S1/2* cells obtained with a Seahorse Xfe 96 Analyzer (mean +/- SEM, n=>12, technical replicates, representative experiment shown). (**F**) Bioenergetic assays of OCR in HAP1 WT and Δ*S1/2* cells in growth medium with formaldehyde (15 μM) and without HT (3 days) (mean +/- SEM, n=>12, technical replicates, representative experiment shown). (**G**) OCR flux measurements using a Resipher of HAP1 WT cells (pre-treated cells 3 days in -HT +/- formaldehyde (15 μM)).

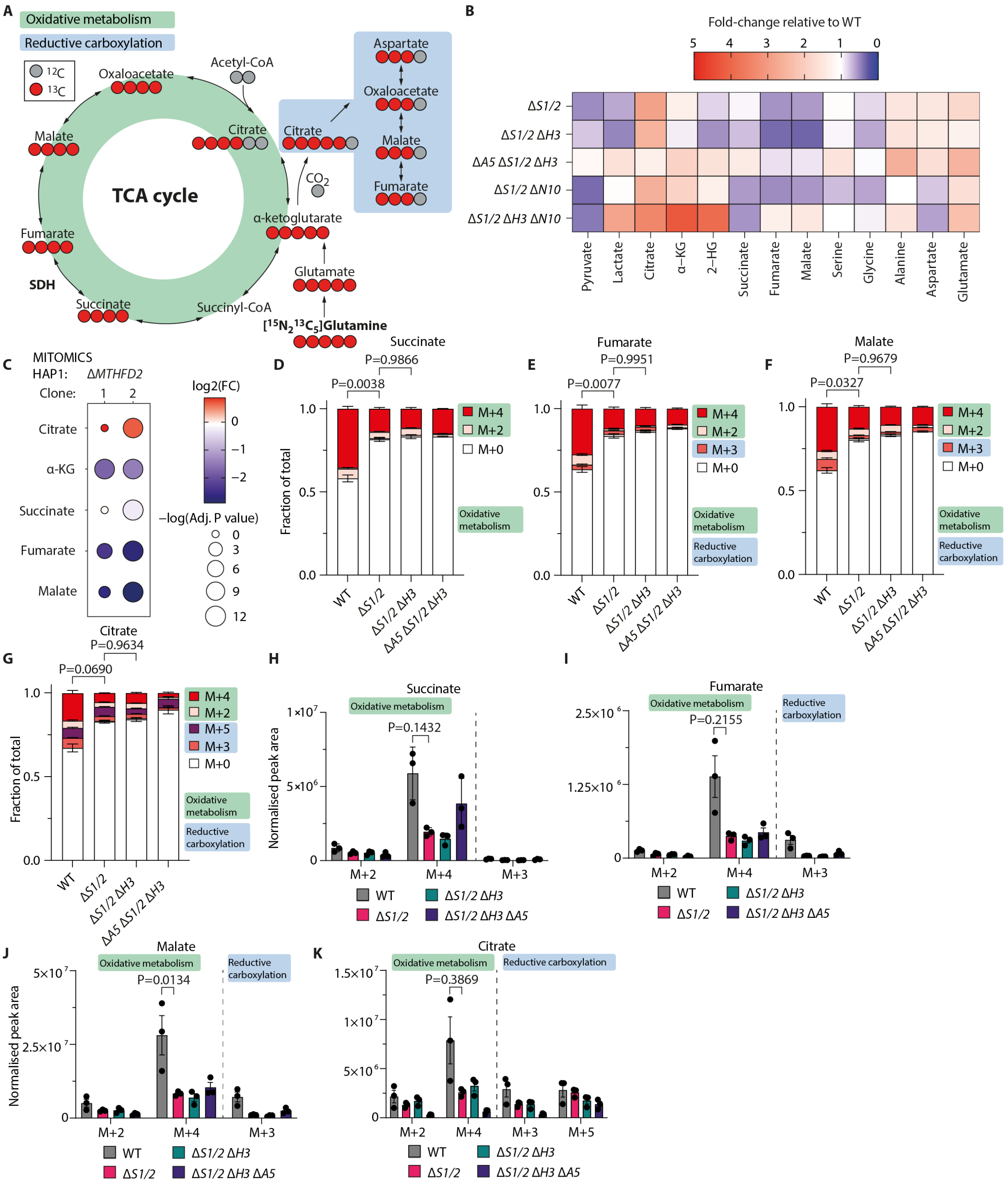

Fig. S6. (**A**) TCA cycle, including metabolic tracing strategy to determine oxidative metabolism and reductive carboxylation contributions. (**B**) Quantification of steady state metabolite levels using GC/MS (mean, n=3). (**C**) Metabolite levels in MTHFD2-deficient HAP1 cells (MITOMICS) (*25*). (**D**) Fractional isotopic labelling (^{13}C) of succinate in HAP1 strains fed with [$^{15}N_2$,$^{13}C_5$]glutamine for 24 hours (mean +/- SEM, n=3). (**E**) Fractional isotopic labelling (^{13}C) of fumarate in HAP1 strains fed [$^{15}N_2$,$^{13}C_5$]glutamine (mean +/- SEM, n=3). (**F**) Fractional isotopic

labelling (^{13}C) of malate in HAP1 strains fed with [$^{15}N_2$,$^{13}C_5$]glutamine for 24 hours (mean +/- SEM, n=3). (**G**) Fractional isotopic labelling (^{13}C) of citrate in HAP1 strains fed [$^{15}N_2$,$^{13}C_5$]glutamine (mean +/- SEM, n=3). (**H**) Normalized peak areas for succinate isotopologues that reflect oxidative metabolism (^{13}C M+2/M+4) (mean +/- SEM, n=3). (**I**) Normalized peak areas for fumarate isotopologues that reflect oxidative metabolism (^{13}C M+2/M+4) and reductive carboxylation (^{13}C M+3) (mean +/- SEM, n=3). (**J**) Normalized peak areas for malate isotopologues that reflect oxidative metabolism (^{13}C M+2/M+4) and reductive carboxylation (^{13}C M+3) (mean +/- SEM, n=3). (**K**) Normalized peak areas for citrate isotopologues that reflect oxidative metabolism (^{13}C M+2/M+4) and reductive carboxylation (^{13}C M+3/M+5) (mean +/- SEM, n=3). P values were determined by by pairwise χ^2 tests of average distributions (D-G) and two-way ANOVA (H-K).

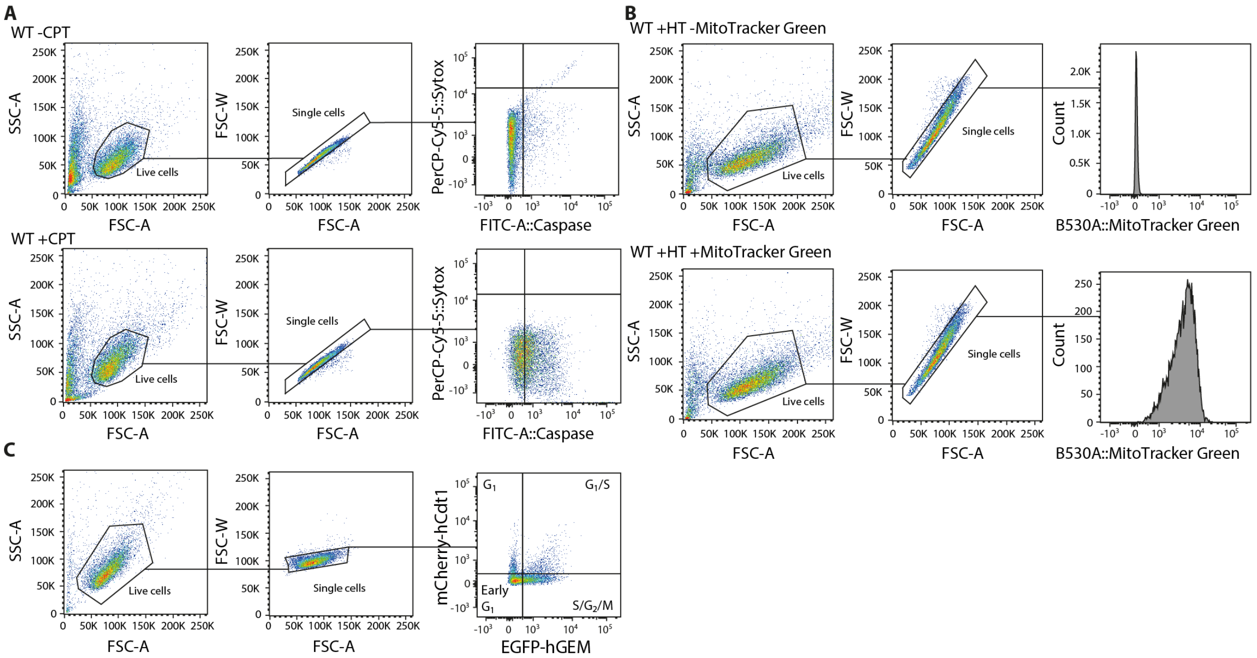

Fig. S7. (**A**) Gating strategies for flowcytometry data of fig. S3A. (**B**) Gating strategies for flowcytometry data of fig. S3B. (**C**) Gating strategies for flowcytometry data of fig. S1H-J and S2H.

Gene	Sequence (5’ → 3’)
Adh5 sgRNA top	ATCTTGGGACATGAAGGTGC
Adh5 sgRNA bottom	CCTCGGGGTCAGCTCCGCTC
ADH5	ATCACTGGAAAACAACCCTC
HDAC3 sgRNA1	GAGCAGAACTCAAAGAGCCC
HDAC3 sgRNA2	TGTCAATGTAGAGCACCCGA
NDUFA10	GGATAATGAATCCCCGCTTC
Shmt2	GGTGGCCCAGACGCAGGCTG
SHMT2	TCAGACTGGGGAAGCAAACA

Table S1.

gRNA sequences

Primer name	Sequence (5’ → 3’)
Whitehead_PCR1 fw	AGGGCCTATTTCCCATGATTCCTT
Whitehead_PCR1 rv	TCAAAAAAGCACCGACTCGG
Whitehead_PCR2 fw	AATGATACGGCGACCACCGAGATCTACACTCTCTTGTGGAAAGGACGAAACACCG
Whitehead_PCR2 rv	CAAGCAGAAGACGGCATACGAGAT**NNNNNNN**GTGACTGGAGTTCAGACGTGTGCTCTTCCGATCTATTTTAACTTGCTATTTCTAGCTCTAAAAC
Whitehead_Sequence_U6	ACACTCTCTTGTGGAAAGGACGAAACACCG
Stanford_PCR1 fw	AGGCTTGGATTTCTATAACTTCGTATAGCATACATTATAC
Stanford_PCR1 rv	ACATGCATGGCGGTAATACGGTTATC
Stanford_PCR2 fw	AATGATACGGCGACCACCGAGATCTACACGCACAAAAGGAAACTCACCCT
Stanford_PCR2 rv	CAAGCAGAAGACGGCATACGAGAT**NNNNNNN**GTGACTGGAGTTCAGACGTGTGCTCTTCCGATCCGACTCGGTGCCACTTTTTC
Stanford_Sequence_U6	AGACTATAAGTATCCCTTGGAGAACCACCTTGTTG

NNNNNNN: index sequence

Table S2.

Oligos used for NGS library generation.

Primer name	Sequence (5’ → 3’)
NDUFA10 fw	GTGCAAACTGCGCTATGGAAT
NDUFA10 rv	CAGGAAAGTGCTTGAAGCCTA
NDUFA1 fw	ATGTGGTTCGAGATTCTCCCC
NDUFA1 rv	CCTGTGGATGTACGCAGTAGC
NDUFA11 fw	GCCGAAGGTTTTTCGTCAGTA
NDUFA11 rv	GGAGGATTGAGTGTGACTCTGT
HDAC3 fw	CCTGGCATTGACCCATAGCC
HDAC3 rv	CTCTTGGTGAAGCCTTGCATA
Actin fw	CTGGGAGTGGGTGGAGGC
Actin rv	TCAACTGGTCTCAAGTCAGTG

Table S3.

Quantitative PCR primer sequences.

Antibody	Source	Identifier	Dilution	Comment
HDAC3	CST	3949	1:1,000	
ADH5	In-house*	N/A	1:1,000	
SHMT1	CST†	12612	1:1,000	HAP1 protein extracts
SHMT1	CST	80715	1:1,000	Non-HAP1 protein extracts
SHMT2	CST	12762	1:1,000	
NDUFA10	Santa Cruz	sc-376357	1:1,000	
Histone H3	CST	4499	1:2,000	
β-actin	Sigma	A2228	1:10,000	
Vinculin	CST	13901	1:1,000	
GAPDH	CST	97166	1:5,000	
Goat anti-mouse-HRP	JIR‡	115-035-146	1:10,000	
Goat anti-rabbit-HRP	JIR	115-035-045	1:10,000	

*: (*4*)

†: Cell Signaling Technology

‡: Jackson ImmunoResearch

Table S4.

Antibodies used in this study.

Supplementary data

Data file S1. MAGeCK output CRISPR-Cas9 screen Fig. 1F (Stanford library)

Data file S2. MAGeCK output CRISPR-Cas9 screen Fig. 1F (Whitehead library)

Data file S3. MAGeCK output CRISPR-Cas9 screen Fig. 3B (Stanford library)
